# Identification, expression, and comparative genomic analysis of the *IPT* and *CKX* gene families in Chinese cabbage *(Brassica rapa* ssp. *pekinensis)*

**DOI:** 10.1186/1471-2164-14-594

**Published:** 2013-08-30

**Authors:** Zhenning Liu, Yanxia Lv, Mei Zhang, Yapei Liu, Lijun Kong, Minghua Zou, Gang Lu, Jiashu Cao, Xiaolin Yu

**Affiliations:** 1 Laboratory of Cell & Molecular Biology, Institute of Vegetable Science, Zhejiang University, 866 Yuhangtang Road, Hangzhou 310058, P.R.China; 2 Laboratory of Horticultural Plant Growth & Quality Regulation, Ministry of Agriculture, 310058, Hangzhou P.R.China

## Abstract

**Background:**

Cytokinins (CKs) have significant roles in various aspects of plant growth and development, and they are also involved in plant stress adaptations. The fine-tuning of the controlled CK levels in individual tissues, cells, and organelles is properly maintained by isopentenyl transferases (IPTs) and cytokinin oxidase/dehydrogenases (CKXs). Chinese cabbage is one of the most economically important vegetable crops worldwide. The whole genome sequencing of *Brassica rapa* enables us to perform the genome-wide identification and functional analysis of the *IPT* and *CKX* gene families.

**Results:**

In this study, a total of 13 *BrIPT* genes and 12 *BrCKX* genes were identified. The gene structures, conserved domains and phylogenetic relationships were analyzed. The isoelectric point, subcellular localization and glycosylation sites of the proteins were predicted. Segmental duplicates were found in both *BrIPT* and *BrCKX* gene families. We also analyzed evolutionary patterns and divergence of the *IPT* and *CKX* genes in the Cruciferae family. The transcription levels of *BrIPT* and *BrCKX* genes were analyzed to obtain an initial picture of the functions of these genes. Abiotic stress elements related to adverse environmental stimuli were found in the promoter regions of *BrIPT* and *BrCKX* genes and they were confirmed to respond to drought and high salinity conditions. The effects of 6-BA and ABA on the expressions of *BrIPT* and *BrCKX* genes were also investigated.

**Conclusions:**

The expansion of *BrIPT* and *BrCKX* genes after speciation from *Arabidopsis thaliana* is mainly attributed to segmental duplication events during the whole genome triplication (WGT) and substantial duplicated genes are lost during the long evolutionary history. Genes produced by segmental duplication events have changed their expression patterns or may adopted new functions and thus are obtained. *BrIPT* and *BrCKX* genes respond well to drought and high salinity stresses, and their transcripts are affected by exogenous hormones, such as 6-BA and ABA, suggesting their potential roles in abiotic stress conditions and regulatory mechanisms of plant hormone homeostasis. The appropriate modulation of endogenous CKs levels by *IPT* and *CKX* genes is a promising approach for developing economically important high-yielding and high-quality stress-tolerant crops in agriculture.

## Background

Cytokinins (CKs) are a class of plant hormones that have significant roles in various aspects of plant growth and development, including apical dominance, shoot or root branching, leaf expansion, lateral bud growth, photosynthesis, seed germination, floral transition, and leaf senescence [1]. Moreover, these hormones participate in biomass distribution [2] and the response to environmental stimuli [3-5]. CKs signals are sensed and transduced by the two-component signal (TCS) system; the availability of CKs in suitable concentrations in the right place and at the right time is necessary for their interaction with a specific receptor. The fine-tuning of hormone levels in individual tissues, cells, and organelles must be properly maintained by the biosynthetic and metabolic enzymes [6]. CKs synthesis in plants is catalyzed by a family of isopentenyl transferases (IPTs) via the methylerythritol phosphate (MEP) and mevalonate (MVA) pathways [3,7,8]. Further studies reveal that ATP/ADP IPTs control the biosynthesis of isopentenyladenine (iP)- and *trans*-zeatin (tZ)-type CKs, whereas tRNA IPTs are responsible for the synthesis of *cis*-zeatin (cZ)-type CKs [3,7]. The irreversible degradation of CKs and their derivatives is catalyzed by CK oxidase/dehydrogenases (CKXs), which are encoded by a small gene family. The substrate specificities of IPTs have been proven to vary, depending on the origin and species [3]. Similarly, CKX enzymes have their own substrate specificity, with various spatial and temporal expression patterns [9].

The *IPT* and *CKX* gene families in various plants have been identified and cloned, including those in *Arabidopsis thaliana*[10,11], *Oryza sativa*[12,13], *Zea mays*[14,15], *Glycine max*[16], *Solanum lycopersicum*[17], *Triticum aestivum*[18], *Hordeum vulgare*[19], *Sorghum bicolor*[19], and *Populus trichocarpa*[20], among others. A putative ATP/GTP binding site (P-loop motif) exists in all of the deduced protein sequences of IPT, whereas CKX protein sequences have FAD- and CK-binding domains. The functions of *IPT* and *CKX* genes have likewise been extensively studied. The overexpression of *AtIPT4* caused CK-independent shoot formation on calli [21]. The overexpression of *ZmIPT2* in *Arabidopsis* produced transgenic plants with phenotypes indicative of CKs overproduction [14]. Transgenic tobacco with *35S-GmIPT1* similarly had the typical phenotypes of CKs overproduction [22]. The enhanced expression of an *IPT* gene designated as *Sho* showed CK-specific effects, including enhanced shooting and reduced apical dominance, as well as delayed senescence and flowering [23]. In *Arabidopsis*, the *ckx3*/*ckx5* double mutant had higher CKs content, which caused the mutants to have more flower primordia, larger flowers, more siliques, and more seeds; the total seed yield of these mutants increased by 55%, as compared with the wild-type [24]. Ashikari et al. [25] conclusively showed that the reduced expression of *Gn1a*, a gene for *OsCKX2*, caused CKs accumulation in rice inflorescence meristems and increased the number of reproductive organs, thereby enhancing the grain yield by 21%. RNAi was more recently used in barley to silence *HvCKX1*, which increased both the grain number and grain weight [26]. The potential use of CK-related genes in seed size and seed mass determination will become an active field of study in the future. Meanwhile, increasing evidence suggests that CKs are involved in stress response [27-29]. Nishiyama et al. [30] proposed a model for the roles of bioactive CKs and antagonistic ABA under different stresses. All CK-deficient plants with reduced levels of different CKs exhibited strong stress-tolerant phenotypes; the majority of *AtIPT* and *AtCKX* genes were repressed by stress and ABA treatments. The *IPT* and *CKX* genes in maize and soybean similarly responded to drought and salt conditions [16,31]. Given that water deficit and high-salinity stresses have become limiting factors for crop and vegetable production and quality, these studies may provide new insights into breeding stress-resistant plants.

*Brassica rapa* is one of the most economically important vegetable crops worldwide. The whole genome sequencing of *B. rapa* (Chiifu-401-42), by The *Brassica rapa* Genome Sequencing Project Consortium (2011), enables us to undertake the genome-wide identification and functional analysis of the gene families related to the morphological diversity and agronomic traits of *Brassica* crops [32]. Furthermore, *B. rapa* serves as a crucial reference for understanding polyploidy-related crop genome evolution because of its agronomic importance and phylogenetic relationships [33].

Ando et al. [34] isolated cDNA fragments of five putative cytokinin synthase genes from *B. rapa* (*BrIPT1*, -*3*, -*5*, -*7*, and −*8*) and examined their expression levels using Northern blot analysis. O’Keefe et al. [35] identified *BrIPT1*, -*3*, and −*5* as well as *BrCKX1*, -*2*, -*3*, and −*5* in rapid-cycling *Brassica*; their expression levels during pod and seed development were examined. However, Brassicaceae genomes have undergone three rounds of whole genome duplication (WGD); these genomes are referred to as 1R, 2R, and 3R, which are equivalent to the γ, β, and α duplication events, and *Brassica* genomes have undergone another whole genome triplication (WGT) after speciation from *Arabidopsis thaliana* at approximately 17–20 MYA (million years ago) [36-38], leading to significantly increased duplicated gene numbers in *B. rapa*. A total of 41,174 protein-coding genes were identified in the *B. rapa* genome, which were roughly 1.5 times as many genes as those found in *A. thaliana* (27,411 genes in TAIR10) [32]. Accordingly, we hypothesized that the number of *BrIPT* and *BrCKX* genes would be greater than that of their *Arabidopsis* counterparts. The main objectives of our study were to identify all the *IPT* and *CKX* genes in *B. rapa*, to analyze the physiological and biochemical properties of their encoded proteins, to explore their gene expression patterns, to discover the mechanisms of their responses to abiotic stresses and exogenous plant hormones, and to identify several potential genes for breeding to increase plant production, quality, and stress-resistance. Furthermore, we intended to gain genome-level insights into the divergence, variation, and evolution of the *BrIPT* and *BrCKX* gene families.

## Results

### Identification and annotation of the *BrIPT* and *BrCKX* genes of *B. rapa*

The *BrIPT* and *BrCKX* genes were identified based on BLAST search results against the BRAD and NCBI databases. A total of 13 *BrIPT* genes and 12 *BrCKX* genes with confirmed conserved domains were obtained from the *B. rapa* genome (Tables 1, 2). The *BrIPT* and *BrCKX* genes were named according to their homologous genes in *A. thaliana*. Each *AtIPT* and *AtCKX* gene corresponded with approximately one to three *BrIPT* and *BrCKX* genes, except for *AtIPT4* and *AtIPT6*.

**Table 1:** ***IPT*gene family in *Brassica rapa *, along with their molecular details and relevant genomic information**

**Gene name**^**a**^	**Locus**^**b**^	**Chr**^**c**^	**Sub- genome**^**d**^	**ORF length**^**e**^**(bp)**	**Deduced polypeptide**^**f**^			**TargetP**^**g**^	**Glycosylation Sites**^**h**^
					**Length (aa)**	**MW (kDa)**	**PI**		
*BrIPT1-1*	Bra004037	A07	MF2	1002	333	37.8	8.91	C/3	0
*BrIPT1-2*	Bra033933	A02	MF1	1095	364	41.6	8.64	C/1	0
*BrIPT2*	Bra034366	A04	MF1	1392	463	52.4	5.89	—	1
*BrIPT3-1*	Bra040431	A04	MF1	1005	334	37.3	8.11	C/4	2
*BrIPT3-2*	Bra007728	A09	LF	999	332	37.6	8.4	C/5	1
*BrIPT5-1*	Bra002204	A10	LF	1005	334	37.9	6.35	—	1
*BrIPT5-2*	Bra023701	A02	MF2	996	331	37.6	5.75	—	1
*BrIPT7-1*	Bra014968	A07	LF	999	332	37.1	8.68	C/5	1
*BrIPT7-2*	Bra028326	A01	MF1	906	301	33.6	8.71	C/3	1
*BrIPT8-1*	Bra037537	A01	MF1	975	324	36.9	8.5	C/2	0
*BrIPT8-2*	Bra001737	A03	MF2	984	327	37.0	9.26	C/4	1
*BrIPT9-1*	Bra006535	A03	MF1	1392	463	52.2	7.51	C/5	3
*BrIPT9-2*	Bra020081	A02	MF2	1395	464	52.2	7.96	C/5	3

^a^Gene Names given to *BrIPTs* in this work.

^b^Locus represented by the *Brassica rapa* genome database.

^c^Chromosomal localization of the *BrIPTs* and *BrCKXs*, sca represents scaffold.

^d^Three sub-genomes of *Brassica rapa* genome.

^e^Length of open reading frame in base pairs.

^f^Length (number of amino acids), molecular weight (kDa), and isoelectric point (pI) of the deduced polypeptide.

^g^Localization predicted with TargetP. M, mitochondria; C, chloroplast; S, secretory pathways; “—”, unknown location. (1 to 5), highest to lowest possibility.

^h^Glycosylation sites are predicted with NetNGly.

**Table 2:** ***CKX*gene family in *Brassica rapa *, along with their molecular details and relevant genomic information**

**Gene name**^**a**^	**Locus**^**b**^	**Chr**^**c**^	**Sub- genome**^**d**^	**ORF length**^**e**^**(bp)**	**Deduced polypeptide**^**f**^			**TargetP**^**g**^	**Glycosylation** **Sites**^**h**^
					**Length (aa)**	**MW (kDa)**	**PI**		
*BrCKX1-1*	Bra000229	A03	MF2	1323	440	49.3	8.71	—	3
*BrCKX1-2*	Bra016928	A04	MF1	1149	382	42.8	7.25	S/5	4
*BrCKX1-3*	Bra004626	A05	LF	4431	1476	162.7	8.47	M/1	12
*BrCKX2-1*	Bra036719	A09	MF2	1509	502	55.5	5.96	S/2	5
*BrCKX2-2*	Bra040677	sca	LF	1518	505	55.6	5.62	S/2	5
*BrCKX3-1*	Bra002777	A10	LF	1101	366	50.0	5.63	C/5	3
*BrCKX3-2*	Bra035640	A02	MF1	1557	518	58.9	5.62	S/5	3
*BrCKX4*	Bra024135	A03	MF1	1575	524	58.0	5.71	S/1	5
*BrCKX5*	Bra015842	A07	LF	1575	524	58.8	5.62	—	2
*BrCKX6*	Bra007743	A09	LF	1350	449	50.5	7.3	M/2	4
*BrCKX7-1*	Bra002371	A10	LF	1578	525	57.9	4.92	—	5
*BrCKX7-2*	Bra020157	A02	MF2	1554	517	57.6	5.2	—	3

^a^Gene Names given to *BrCKXs* in this work.

^b^Locus represented by the *Brassica rapa* genome database.

^c^Chromosomal localization of the *BrIPTs* and *BrCKXs*, sca represents scaffold.

^d^Three sub-genomes of *Brassica rapa* genome.

^e^Length of open reading frame in base pairs.

^f^Length (number of amino acids), molecular weight (kDa), and isoelectric point (pI) of the deduced polypeptide.

^g^Localization predicted with TargetP. M, mitochondria; C, chloroplast; S, secretory pathways; “—”, unknown location. (1 to 5), highest to lowest possibility.

^h^Glycosylation sites are predicted with NetNGly.

The *BrIPT* genes were distributed on 7 out of 10 chromosomes (except for A05, A06, and A08), belonging to the three sub-genomes (LF, MF1, and MF2). The ORF lengths of the *BrIPT* genes ranged from 906 bp to 1395 bp, which encoded polypeptides of 301 aa to 464 aa with predicted molecular weights ranging from 33.6 kD to 52.4 kD. The theoretical pI ranged from 5.75 to 9.26. The *BrIPT* genes were all predicted to be localized in the chloroplasts. Similarly, the *BrCKX* genes were also distributed on 7 out of 10 chromosomes (except for A01, A06, and A08). The ORF lengths of the *BrCKX* genes were longer than those of the *BrIPT* genes; these ORFs ranged from 1101 bp to 4431 bp and encoded polypeptides of 366 aa to 1476 aa with predicted molecular weights ranging from 42.8 kD to 162.7 kD. The theoretical pI ranged from 4.92 to 8.71. The predicted subcellular localization of the *BrCKX* genes varied and included the chloroplast, mitochondria, and secretory pathways. Protein glycosylation contributes to regulation of enzymatic activity, translocation, and protein stability; thereby, adding an additional and important level of complexity to *CKX* regulation [39]. Similar to the *ZmCKX* genes, the *BrCKX* genes possessed 2 to 12 predicted glycosylation sites.

### Gene structure and conserved domain analysis of *BrIPT* and *BrCKX* genes

**Figure 1:**
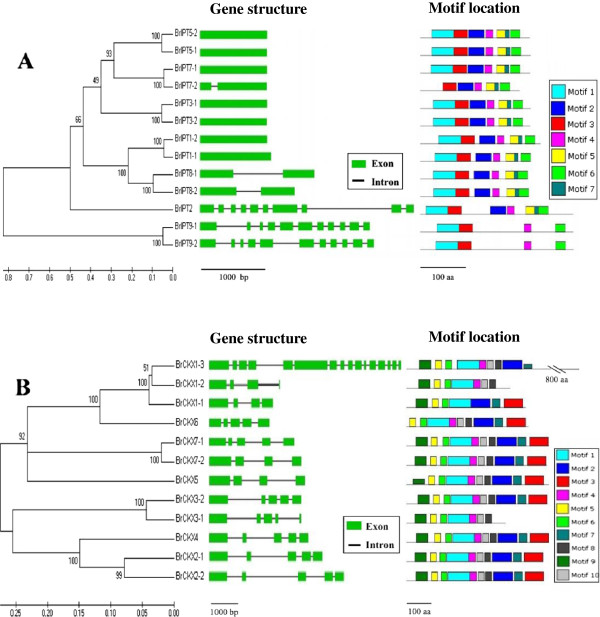
**Gene structure and motif analysis of the *BrIPT *(A) and *BrCKX *(B) genes.** Exons and introns were organized with green boxes and black lines, respectively. Motifs locations were shown with combined block diagrams.

To analyze the structural characteristics and conserved regions of the *BrIPT* and *BrCKX* gene families, their gene structures with exons and introns were mapped, their conserved regions and motifs were examined, and their putative protein sequences were aligned. Similar to the *IPT* gene families in other plants, *BrIPT* genes could be grouped into two types of *IPT* genes: plant adenylate IPT- or ADP/ATP-dependent genes (*BrIPT1*, *-3*, *-5*, *-7* and −*8*) and plant tRNA IPT- or tRNA-dependent genes (*BrIPT2* and *BrIPT9*). Plant tRNA IPT genes could be further divided into plant tRNA *IPT* genes of eukaryotic (*BrIPT2*) and prokaryotic (*BrIPT9*) origin. Seven plant adenylate *BrIPT* genes were notably composed of a single exon without introns and three plant adenylate *BrIPT* genes only had one intron. By contrast, the longer plant tRNA *BrIPT* genes had at least ten exons and nine introns, which was consistent with the more complicated tRNA *IPT* gene structures in other plants (Figure 1A). The multiple alignment of AtIPT protein sequences showed that AtIPT2 contained two inserted regions of approximately 20–80 amino acids in length; while the carboxyl-terminal region of the said protein sequences had an extra 40 amino acids. A similar structure was observed in BrIPT2. AtIPT9 and BrIPT9 had five shorter discontinuous inserted regions of approximately 8–14 amino acids instead of the two larger regions in AtIPT2 and BrIPT2. However, the carboxyl-terminal region with an extra 40 amino acids was conserved in both BrIPT2 and BrIPT9 (Additional file 1). Two types of *IPT* gene structures are closely related and had also unique conserved motifs. Seven conserved motifs were found for the BrIPT protein sequences (Figure 1A, Additional file 2). All BrIPT protein sequences contained one or two conserved regions that belonged to the P-loop NTPase superfamily (cl09099). Furthermore, three plant tRNA BrIPT protein sequences contained either PLNO2748 or PLNO2840 multi-domains, which were unique to tRNA IPTs. The multiple alignment of BrIPT protein sequences with AtIPT protein sequences likewise revealed two corresponding conserved regions, which were designated as Region a and Region b. Region a was found in all the BrIPT protein sequences; it was identified as a putative ATP/GTP-binding site (P-loop) motif, with a core sequence of TGxGKS (Motif 1). Region b was only present in three plant tRNA BrIPT protein sequences; this region was classified as a tRNA binding site. The two types of plant tRNA BrIPT proteins had different consensus sequences, which were denoted by a red box in Additional file 1.

**Figure 2:**
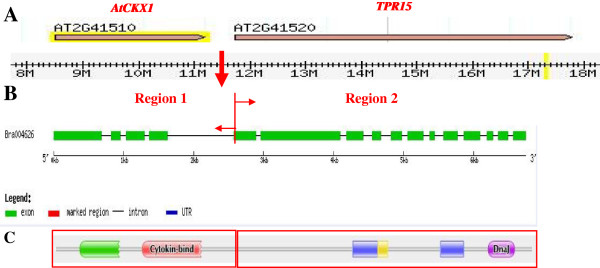
**Gene structure and conserved domain analysis of *BrCKX1-3.*** Physical positions of the *AtCKX1* and *AtTPR15* on the chromosome of *A. thaliana ***(A)**. Gene structure of *BrCKX1-3 ***(B)**. Conserved domains of *BrCKX1-3 ***(C)**.

The gene structures of the *BrCKX* genes were more diverse and contained several exons and introns (Figure 1B). A FAD-binding domain and a CK-binding domain were generally indispensable in a functional *CKX* gene; these domains belonged to the FAD-binding (cl14794) and CK-binding (cl07775) superfamilies, respectively. The unique *BrCKX1-3* gene had a longer sequence length and more complex conserved domains (Figure 2). Based on the alignment and conserved domains analysis, the multi-domain *BrCKX1-3* was composed of two regions, Region 1 and Region 2. Region 1 contained the typical conserved domains of the *CKX* gene family, namely, the FAD- and CK-binding domains. By contrast, Region 2 had the characteristics of a TPR protein and contained a TPR repeat and a Dnaj domain. However, the *AtCKX1 (AT2G41510*) and *AtTPR15* (*AT2G41520*) genes in *Arabidopsis* are two completely different functional genes, even though these genes are located next to each other. Based on the gene structures analysis, as compared with that of *A. thaliana*, we proposed that *BrCKX1-3* was formed by the fusion of the *CKX* and *TPR* genes. However, the actual mechanism of *BrCKX1-3* formation is still unknown. Ten conserved motifs were found in the *BrCKX* genes (Figure 1B, Additional file 3). The sequence alignment showed that the FAD-binding domain was well conserved in the N-terminal halves of all known *CKX* sequences (Additional file 4). Moreover, the GHS motif (Motif 5) was perfectly conserved in this family protein sequences. We also identified several additional conserved motifs, including the VPHPWLNL motif (Motif 2) at position 450 and a PGQxIF signature (Motif 3) at the C-terminal end of the CKX protein sequences. These motifs appeared to be specific to CKX enzymes.

### Chromosomal mapping and duplications of *BrIPT* and *BrCKX* genes

**Figure 3:**
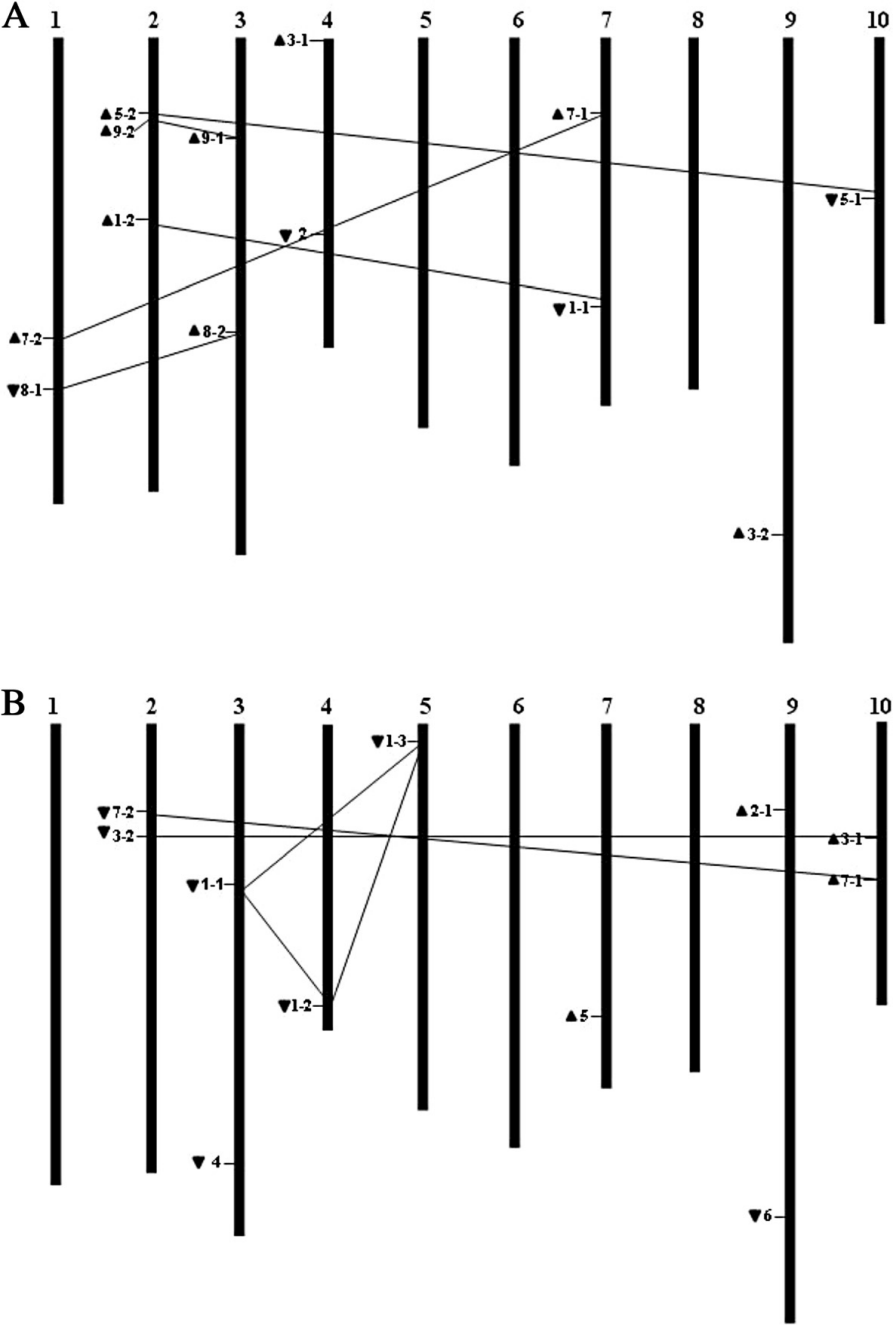
**Chromosomal mapping of *BrIPT *(A) and *BrCKX *(B) genes.** The arrows next to gene names show the direction of transcription. *BrCKX2-2* is not mapped for its location on scaffold000232. Segmental duplicates are connected by inter-chromosomal lines.

We mapped *BrIPT* and *BrCKX* genes on chromosomes (Figure 3A, 3B). *BrCKX2-2* was not mapped due to its location on scaffold000232. Even genes with high similarity were not located on the same chromosome. Segmental and tandem duplications are important sources of gene duplication. Gu et al. [15] proposed that the *CKX* gene expansion occurred mainly through segmental duplications in maize, rice, and poplar. In this study, no tandem duplicated gene pairs were found. However, segmental duplicates existed in both *BrIPT* and *BrCKX* gene families (Additional file 5 and Additional file 6). Five pairs of segmental duplicates were found in 13 *BrIPT* genes: *BrIPT1-1* (A07) and *BrIPT1-2* (A02); *BrIPT5-1* (A10) and *BrIPT5-2* (A02); *BrIPT7-1* (A07) and *BrIPT7-2* (A01); *BrIPT8-1* (A01) and *BrIPT8-2* (A03); and *BrIPT9-1* (A03) and *BrIPT9-2* (A02). Three pairs of segmental duplicates were found in 12 *BrCKX* genes: *BrCKX1-1* (A03), *BrCKX1-2* (A04) and *BrCKX1-3* (A04); *BrCKX3-1* (A10) and *BrCKX3-2* (A02); and *BrCKX7-1* (A10) and *BrCKX7-2* (A02). Each pair of segmental duplicates was distributed on different chromosomes. *BrIPT3-1* and *BrIPT3-2* were notably not considered as a pair of segmental duplicates, even though they shared high sequence similarity.

### Phylogenetic relationships of the *BrIPT* and *BrCKX* gene families

**Figure 4:**
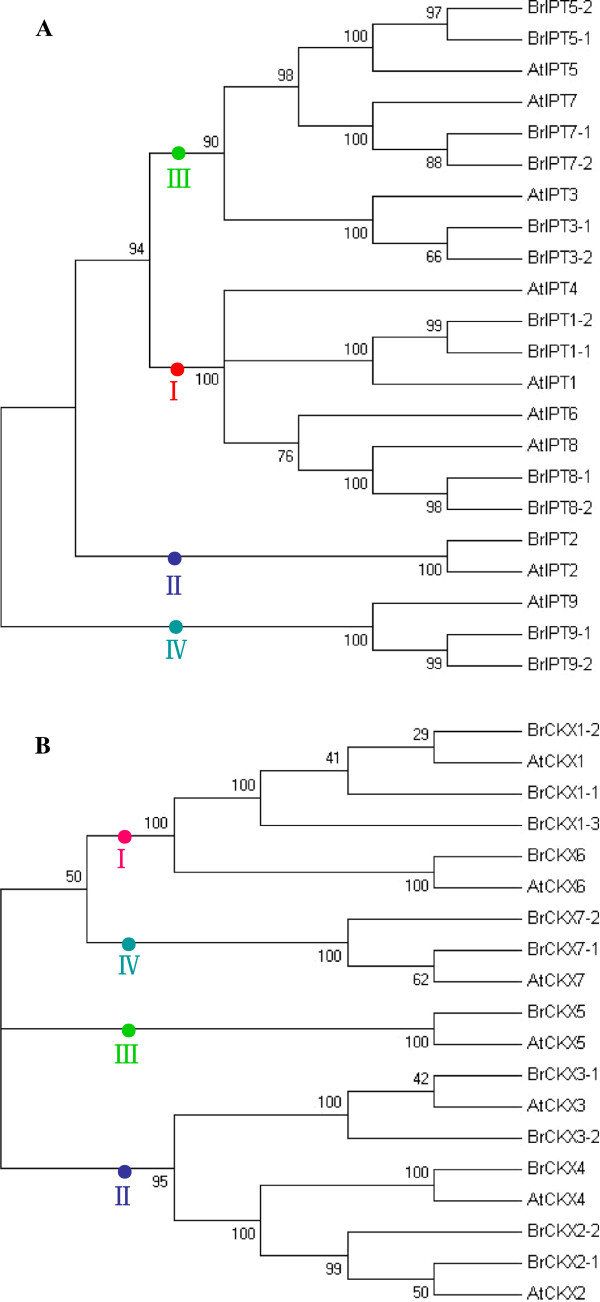
**Phylogenetic relationships of *BrIPTs *and *BrCKXs, *together with their *Arabidopsis *conterparts, respectively.** Trees were constructed using NJ method by the MEGA (version 5.0) program. Both *BrIPTs***(A)** and *BrCKXs***(B)** were divided into four groups indicated by differently colored dots.

**Figure 5:**
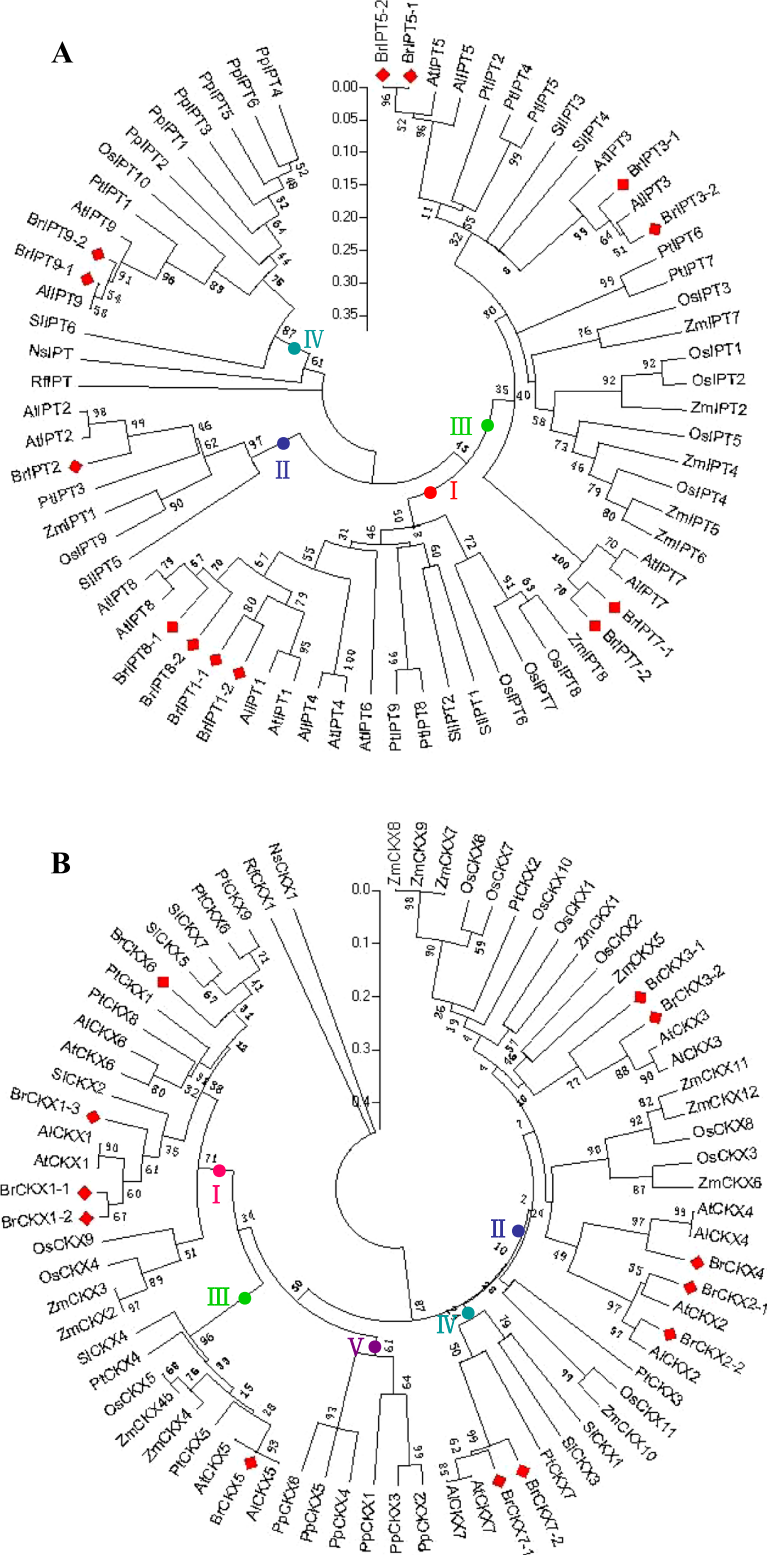
**Phylogenetic relationships of *IPT *and *CKX *gene families from several species.** Trees were constructed using NJ method by the MEGA (version 5.0) program. The phylogenetic tree of *IPT* gene family contained *A. thaliana* (9), *A. lyrata* (7), *B. rapa* (13), *O. sativa* (10), *Z. mays* (8), *S. lycopersicum* (6), *P. trichocarpa* (9), and *P. patens* (6), as well as *NsIPT* and *RfIPT***(A)**. The *IPT* genes were divided into four groups indicated by differently colored dots. *BrIPT* genes were highlighted by red rectangles. The phylogenetic tree of *CKX* gene family contained *A. thaliana* (7), *A. lyrata* (7), *B. rapa* (12), *O. sativa* (11), *Z. mays* (13), *S. lycopersicum* (6), *P. trichocarpa* (9), and *P. patens* (6), as well as *NsCKX1* and *RfCKX1***(B)**. The *CKX* gene family was divided into five groups indicated by differently colored dots. *BrCKX* genes were highlighted by red rectangles.

To identify subgroups and reveal the evolutionary relationships of the two gene families, the phylogenetic analyses of the *BrIPT* and *AtIPT* genes, as well as that of the *BrCKX* and *AtCKX* genes, were performed with the putative protein sequences using MEGA (version 5.0) software. As shown in Figure 4A, the *BrIPTs* and *AtIPTs* were clustered into four subgroups: Group I (*BrIPT1-1, -1-2,* -8-1 and −*8-2*), Group II (*BrIPT2*), Group III (*BrIPT3-1, -3-2, -5-1, -5-2, -7-1* and −*7-2*), and Group IV (*BrIPT9-1* and *BrIPT9-2*). Group I and Group III included the majority of the *BrIPT* genes, which belonged to the plant adenylate *IPT* genes. The tRNA-dependent *BrIPT* genes and ADP/ATP-dependent *BrIPT* genes were highly diverse; these genes could be divided into two different subgroups. *BrIPT2* in Group II, the *AtIPT2* homolog, is a plant tRNA *IPT* gene of eukaryotic origin. *BrIPT9-1*, *BrIPT9-2,* and *AtIPT9* all shared the properties of plant tRNA *IPT* genes with prokaryotic origins, and were classified into Group IV. Similarly, the *BrCKX* and *AtCKX* genes were also clustered into four subgroups: Group I (*BrCKX1-1*, *-1-2*, *-1-3* and −*6*), Group II (*BrCKX2-1*, *-2-2, -3-1*, *-3-2* and −*4*), Group III (*BrCKX5*), and Group IV (*BrCKX7-1* and *BrCKX7-2*) (Figure 4B). Further phylogenetic reconstructions using IPT and CKX protein sequences from various species, including dicotyledons, monocotyledons, moss, algae, and bacteria, were performed to confirm the *IPT* and *CKX* genes subgroups, as well as to investigate the evolution of *BrIPT* and *BrCKX* genes. The corresponding plant *IPT* genes were divided into four groups: Groups I, II, III, and IV (Figure 5A). As shown in the figure, genes in dicotyledons and monocotyledons were randomly dispersed among the four groups. This phenomenon suggested that *IPT* genes originated from both dicotyledons and monocotyledons or were not lost after the divergence of the two plant groups. Furthermore, only the six *IPT* genes in the bryophyte *P. patens* (*PpIPT*) were found in Group IV, which also included the plant tRNA *IPT* genes of prokaryotic origin, implying that *PpIPT* genes possiblely originated from the tRNA IPT genes. Plant *CKX* genes were classified into five groups, with Group V as an additional group (Figure 5B). Group V contained only the six *PpCKX* genes.

### Evolutionary patterns and divergence of *IPT* and *CKX* genes in the Cruciferae family

**Figure 6:**
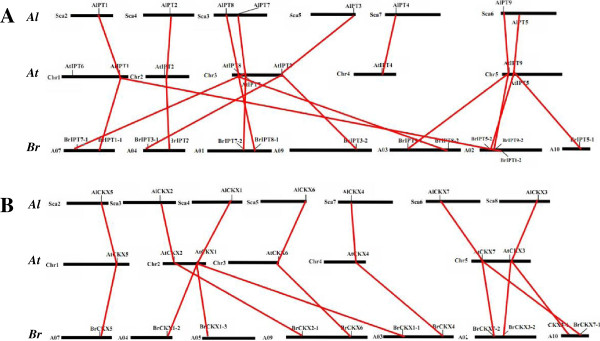
**Relationship diagram of homologous *IPT *(A) and *CKX *(B) genes chromosomal distribution among*B. rapa *(*Br*), *A. thaliana *(*At*), and*A. lyrata*(*Al*).** Chromosomes are indicated by black lines with different lengths according to their own sizes. Homologous genes are linked by red lines.

To elucidate the evolutionary patterns and divergence of *IPT* and *CKX* genes in Cruciferae family, the three sequenced cruciferous species (*B. rapa*, *A. thaliana*, and *A. lyrata*) were chosen to make a genome-level comparison. The genome size of the North American *A. lyrata* is 207 Mb, with eight chromosomes [40]. *A. thaliana*, with its 125 Mb genome and five chromosomes, has undergone a ~30% reduction in genome size [41] and nine to ten chromosomal rearrangements [42,43]. *A. thaliana* was estimated to have diverged from *A. lyrata* approximately 10 MYA [44-46]. *B. rapa* has approximately 529 Mb and ten chromosomes; this species is believed to have emerged from the *Brassica* progenitor approximately 8 MYA [33]. As shown in Figure 6A, 6B, the *IPT* and *CKX* genes were well dispersed on the chromosomes, except for the Sca1 chromosome in *A. lyrata* as well as A01, A06, and A08 in *B. rapa*. The corresponding relationships of the *IPT* and *CKX* genes on chromosomes were clearly evident: Sca2 corresponded to Chr1; Sca3 and Sca4 corresponded to Chr2; Sca3 and Sca5 corresponded to Chr3; Sca7 corresponded to Chr4; Sca6 and Sca8 corresponded to Chr5. This trend was consistent with a previous comparison of the *A. lyrata* and *A. thaliana* genomes [40]. Traces of chromosomal rearrangement and simplification in the *A. thaliana* genome were easily captured by the analysis. The corresponding relationships between *B. rapa* and *A. thaliana* were still regular, although relatively more complicated. Homologous genes were separately existed on Chr1 as paired with A02 and A07, Chr2 as paired with A03, A04, and A09, Chr3 as paired with A04, A07, and A09, Chr4 as paired with A03, and Chr5 as paired with A02, A03, and A10. This trend was consistent with the segmental collinearity of the *B. rapa* and *A. thaliana* genomes [47].

**Figure 7:**
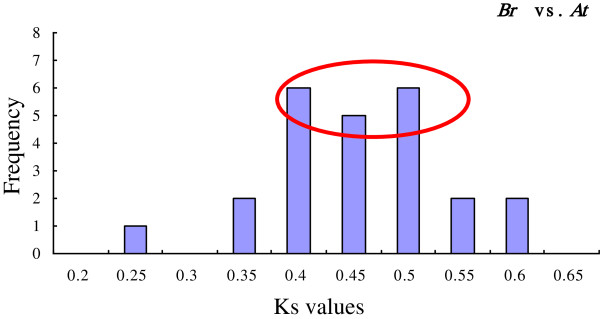
**The *K***_**s **_**values distribution of *IPT *and *CKX *genes in the genomes of *B. rapa *and *A. thaliana.*** The vertical axis indicates the frequency of paired sequences, whereas the horizontal axis denotes the *K*_s_ values with an interval of 0.05. The bars depict the positions of the modes of *K*_s_ distributions obtained from orthologous gene pairs. The peaks of the bars are marked with a red circle which represents the principal distribution extent of the *K*_s_ values.

To reveal the divergence of *IPT* and *CKX* genes in the Cruciferae family, the *K*_s_ and *K*_a_ modes for the paralogs and orthologs in the three genomes were compared (Additional file 7 and Additional file 8). Evidently, the *K*_s_ values of the paralogs in *B. rapa* were much smaller than the values of the orthologous genes between *B. rapa*, *A. thaliana*, and *A. lyrata*. The *K*_s_ values obtained from comparisons of the sets of putative orthologs were used to calculate the divergence time between *B. rapa* and *A. thaliana*. The *K*_s_ values ranged from 0.25 to 0.6, with a concentrated location between 0.4 and 0.5 (Figure 7). The calculation of divergence time was based on the neutral substitution rate of 1.5×10^-8^ substitutions per site per year for *Chs*[48]. Our results showed *B. rapa* diverged from *A. thaliana* at around 26–33 MYA, which coincided well with previous studies of speciation between the *Brassica* and *Arabidopsis* genomes [46,49-51].

### Tissue or organ-specific expression of *BrIPT* and *BrCKX* genes

**Figure 8:**
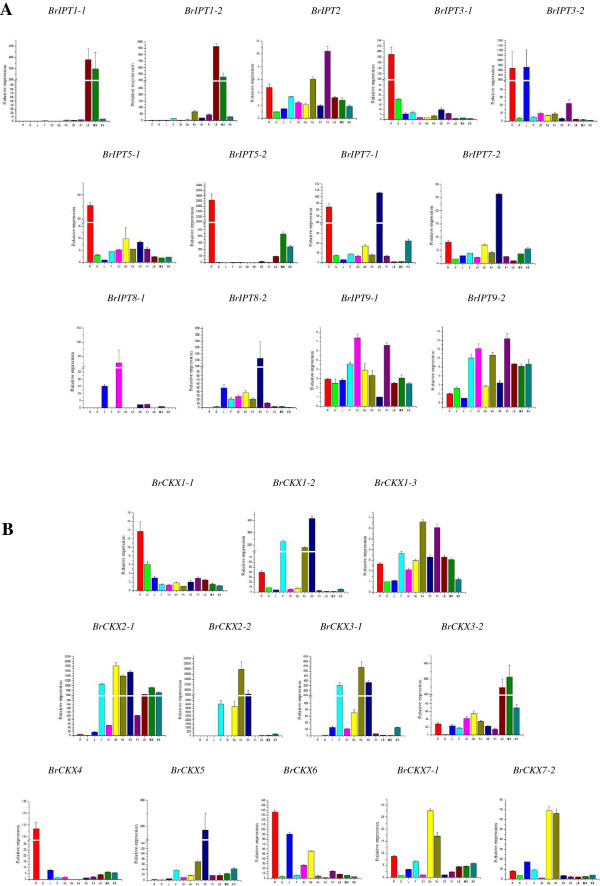
**Relative expression profiles of *BrIPT*s (A) and *BrCKX*s (B) in various tissues and organs.** R, roots; S, floral stems; L, leaves; F, flowers; Si, immature siliques; Se, sepals; Pe, petals; St, stamens; Pi, pistils; LB, little buds; MB, medium-sized buds; and BB, big buds. Error bars represent ±SE of two biological replicates.

Tissue-specific and developmental stage-related expression data are useful in the identification of genes that are involved in defining the precise nature of individual tissues in a given developmental stage [16]. CKs have central regulatory functions during plant development and have essential functions during the adaptation to adverse environments. A clear expression profile of the genes of interest is an indispensable step to find and utilize agriculturally important genes. The homeostasis of the CK level in various tissues and organs is mainly regulated by the IPT and CKX enzymes. Thus, to obtain an initial picture of the functions of *BrIPT* and *BrCKX* genes during the vegetative and reproductive development, the transcript levels of these genes in the roots, stems, leaves, flowers, immature siliques, sepals, petals, stamens, pistils, little buds, medium-sized buds, and big buds were analyzed by qRT-PCR. As shown in Figure 8A, the tRNA *IPT* genes, *BrIPT2*, *BrIPT9-1*, and *BrIPT9-2*, were ubiquitously expressed, which was similar to the expression patterns of *AtIPT2* and *AtIPT9*[7]. Other *BrIPT* genes demonstrated tissue or organ-specific expression levels. The expression of the *BrIPT* genes was similar to that of the *AtIPT* genes. *BrIPT1-1* and *BrIPT1-2* were highly expressed in the little and medium-sized buds, but reached very low levels in the big buds. *BrIPT3-1* and *BrIPT3-2* were highly expressed in the roots, and *BrIPT3-2* was also expressed with very high levels in the leaves. *BrIPT5-1* and *BrIPT5-2* were mainly expressed in the roots. *BrIPT7-1* and *BrIPT7-2* were primarily transcribed in the stamens and roots. These segmental duplicated genes had uniform expression levels. *BrIPT8-1* and *BrIPT8-2* had unique expression patterns. *BrIPT8-1* had the highest expression levels in siliques, whereas *BrIPT8-2* was mainly expressed in stamens. The *BrIPT* genes were expressed abundantly in the reproductive organs. Maybe the roots alone could not supply sufficient CKs for the increasing demand in the reproductive organs. As can be seen from Figure 8B, the expression of *BrCKX* genes was more diverse, only *BrCKX7-1* and *BrCKX7-2* were uniformly expressed at high levels in sepals and petals. *BrCKX1-1* was mainly expressed in the roots; *BrCKX1-2* maintained a high level in the stamens, flowers, and petals. *BrCKX1-3* seemed to be ubiquitously expressed, with relatively higher levels in the petals and pistils. *BrCKX2-1* and *BrCKX2-2* were mainly expressed in the reproductive organs, including the flowers, sepals, petals, and stamens. However, the expression of both genes was barely detected in vegetative organs. *BrCKX3-1* was highly expressed in the petals, stamens, and flowers whereas *BrCKX3-2* was mainly expressed in the floral buds. *BrCKX4* and *BrCKX5* had extremely high levels in the roots and stamens, respectively. *BrCKX6* mostly transcribed in the roots, leaves, and sepals. The segmental duplicated pairs of *BrIPT* genes had similar expression profiles. However, this was the case in only one of the four segmental duplicated pairs of *BrCKX* genes. CKs could be rapidly and precisely transported from the synthetic positions to the functional parts via a multiple of putative purine permeases (PUPs) [52,53] and equilibrative nucleotide transporters (ENTs) [54,55] involved in CKs transport in plants. Thus, CKX enzymes are compulsory to be present in various organs to maintain the finely-tuned homeostasis of CKs levels. Although the segmental duplicated gene pairs originated from common ancestral genes after the WGT, they may have probably evolved into independent new genes with irreplaceable functions.

### Stress-inducible *cis*-regulatory elements in the promoter regions of *BrIPT* and *BrCKX* genes

The *cis*-regulatory elements, which are located in the upstream regions of genes, act as the binding sites for TFs; thus, these sequences have essential roles in determining the tissue-specific or stress-responsive expression patterns of genes [16]. Increasing evidence has showed that genes responsive to multiple stimuli are closely correlated with *cis*-regulatory elements in their promoter regions [56,57]. To further understand transcriptional regulation and the potential functions of the *BrIPT* and *BrCKX* genes, putative promoter regions in the 2000 bp sequences upstream of the transcriptional start site were used to identify putative stress-responsive *cis*-regulatory elements. Several abiotic stress elements were found (Additional file 9 and Additional file 10). One salt-stress (S000453), one heat-stress (S00030), one cold-stress (S000407), one wound-stress (S000457), and three drought-stress (S000176, S000408, and S000415) *cis*-elements were common in the promoter regions of the *BrIPT* and *BrCKX* genes. Therefore, *B. rapa* may adapt to stressful environments by changing its CKs content. Particularly, *BrIPT3-1*, *BrIPT3-2*, and *BrCKX3-2* had up to 32, 26, and 24 cold-stress elements (S000407), respectively. The number of drought-stress elements (S000415) in *BrCKX5* reached 28. The other remaining genes likewise possessed abundant stress-inducible *cis*-regulatory elements. The tRNA *IPT* genes in *Arabidopsis* were notably unaffected by the mineral nutrients, auxin, and CK concentrations [7]. By contrast, three tRNA *IPT* genes in *B. rapa*, *BrIPT2*, *BrIPT9-1*, and *BrIPT9-2* were also rich in the five types of *cis*-regulatory elements based on our results. Therefore, we hypothesized that the tRNA *IPT* genes might also have important functions in the adaptation to adverse environmental stimuli. Moreover, 0.4 and 1.2 abiotic stress-inducible *cis*-elements per promoter were found in the *GmIPT* and *GmCKX* genes, respectively [16]. Similarly, *BrCKX* genes had relatively more stress-responsive elements than *BrIPT* genes.

### Expression profiles of *BrIPT* and *BrCKX* genes under drought and salt stress

**Figure 9:**
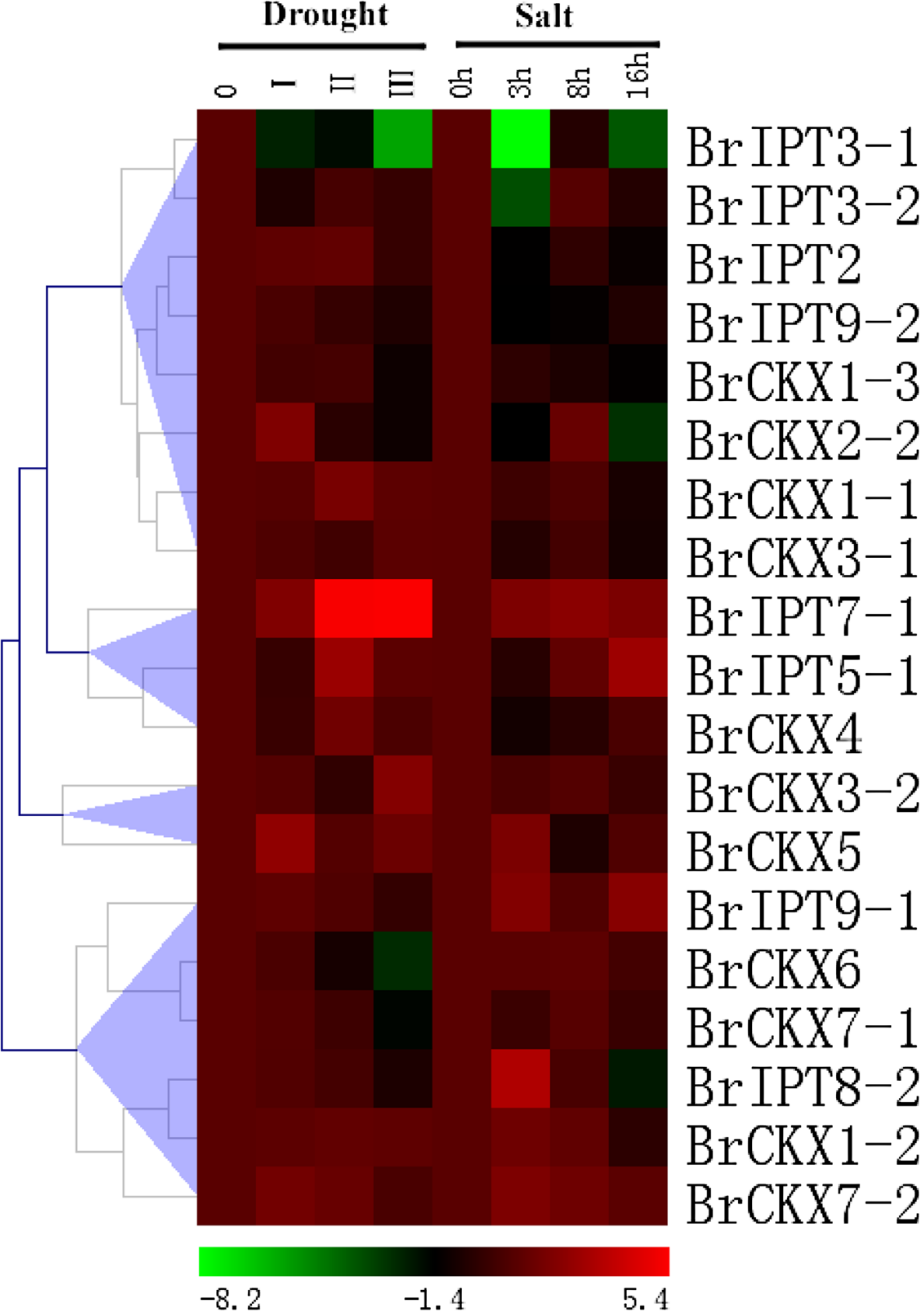
**The heat map shows the real-time quantitative RT-PCR (qRT-PCR) analysis results of *BrIPT *and *BrCKX *genes under drought and salt treatments with two biological and three technical replicates.** The expression levels of genes are presented using fold-change values transformed to Log_2_ format compared to control. The Log_2_ (fold-change values) and the color scale are shown at the bottom of heat map.

Previous studies revealed that the CKs content was correlated with stress-resistance; changed transcripts of *IPT* and *CKX* genes were observed in plants when they were exposed to drought and high salinity conditions, including *Arabidopsis*[30], rice [10], maize, [31] and soybeans [16]. Theoretically, the *BrIPT* and *BrCKX* genes could also respond to various abiotic stresses because of the presence of stress-inducible *cis*-regulatory elements in their promoter regions. qRT-PCR was used to analyse the expression profiles of *BrIPT* and *BrCKX* genes under drought and salt stress conditions. The data were presented with clusters using fold-change values transformed to Log_2_ format. As shown in Figure 9, in drought conditions, the majority of the *BrIPT* and *BrCKX* genes were initially induced and upregulated before falling to basal levels during the time-course, despite the continuous application of the stressor. To be more specific, *BrIPT7-1* and *BrCKX3-2* were continuously induced and maintained at high levels in conditions of serious drought. *BrIPT7-1* even showed remarkably more than a 40-fold increase in its expression levels. Three *CKX* genes, *BrCKX1-1*, *BrCKX1-2*, and *BrCKX5* maintained their relatively high expressions throughout the experiment. Notably, in level III, genes such as *BrIPT2*, -3-1, -5-1, -*9-1* and −*9-2*, as well as *BrCKX4* and −*7-2*, eventually returned to the basal level, expressions of *BrIPT8-2, BrCKX1-3* and *BrCKX7-1* dropped below the basal level, transcripts of *BrIPT3-1* and *BrCKX2-2* were even hardly detectable. By contrast, salt treatment induced variable expression patterns. Still, the majority of *BrIPT* and *BrCKX* genes were primarily induced and upregulated. However, their transcripts fell with prolonged treatment time. The transcription of *BrIPT9-2* seemed to be unaffected. Only *BrIPT5-1* was continuously induced with a 7-fold increase in its expression levels, whereas *BrCKX1-3* was suppressed and its expression levels dropped throughout the experiment. *BrIPT7-1* and −*9-1*, as well as *BrCKX4*, -*6* and −*7-1*, had relatively high levels after 16 h of salt treatment while several genes returned to the basal level, namely, *BrCKX1-2*, -*3-2*, -*5*, -*6*, and −*7-2*. Moreover, transcripts of *BrIPT2*, *BrCKX1-1*, *BrCKX3-1*, *BrIPT3-1*, *BrIPT8-2*, and *BrCKX2-2* dropped below the basal level and were eventually undetectable at this time point. Except for a similar response to drought and salt treatments for both *BrIPT* and *BrCKX* genes, we noted that the transcripts of both *BrIPT3-1* and *BrCKX2-2* had almost disappeared with treatment time going on. Consistent with the results in maize [31], the tRNA *IPT* genes in *Brassica* responded to stressful environments as well.

### Effects of exogenous 6-BA and ABA on the expressions of *BrIPT* and *BrCKX* genes

**Figure 10:**
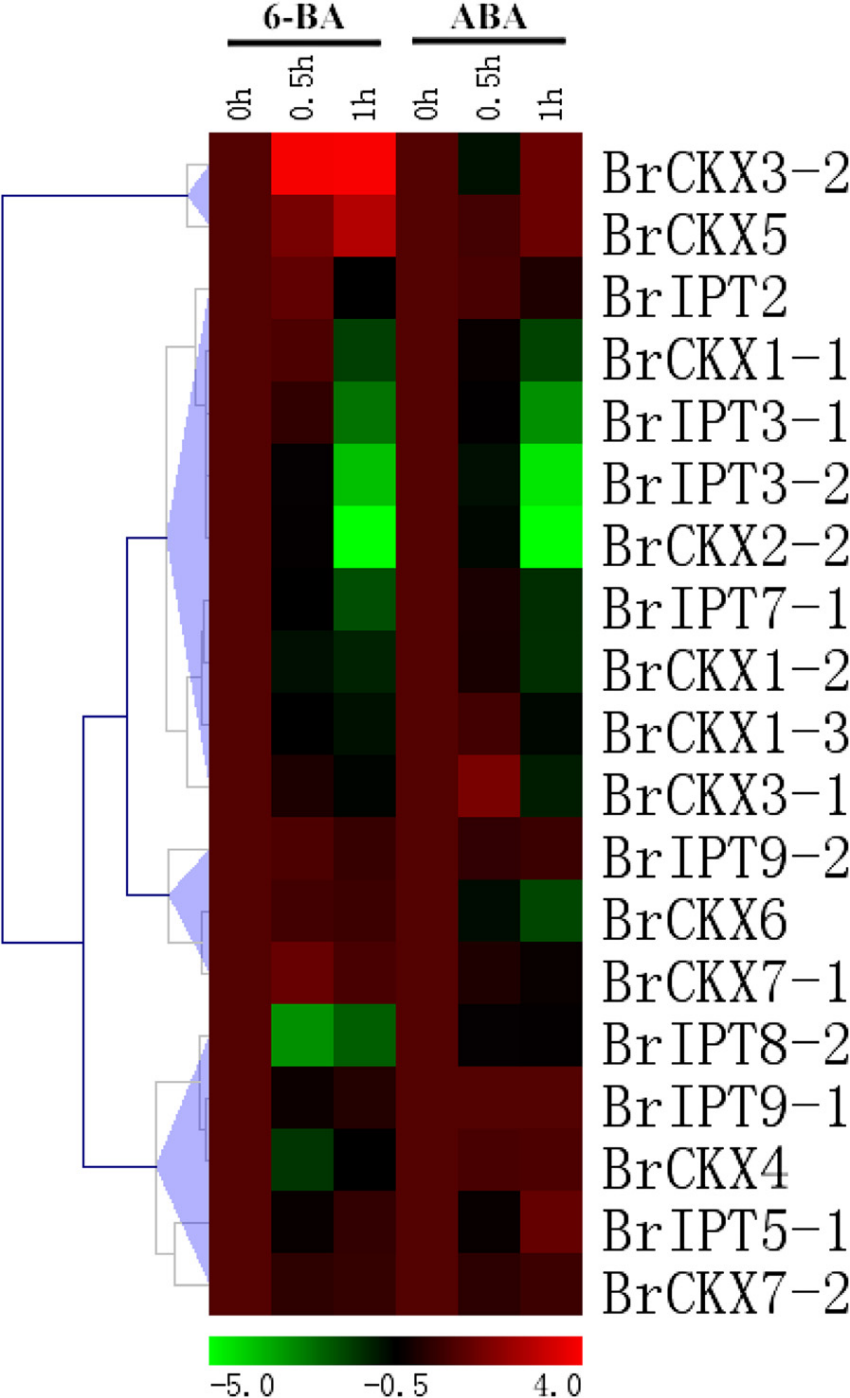
**The heat map shows the real-time quantitative RT-PCR (qRT-PCR) analysis results of *BrIPT *and *BrCKX *genes with exogenous 6-BA and ABA treatments with two biological and three technical replicates.** The expression levels of genes are presented using fold-change values transformed to Log_2_ format compared to control. The Log_2_ (fold-change values) and the color scale are shown at the bottom of heat map.

Various phytohormones regulate the abiotic and biotic stress resistance. CKs and ABA are two important plant hormones involved in stress response. CKs and ABA have been postulated to exert antagonistic activities during the adaptation to environmental stress in plants [4,58,59]. To determine the effects of exogenous CKs and ABA on the expressions of *BrIPT* and *BrCKX* genes, we investigated the expression profiles of these genes using qRT-PCR analyses and data were also presented with clusters using fold-change values transformed to Log_2_ format. Viewing from Figure 10, five adenylate *IPT* genes in *B. rapa* were significantly repressed at 0.5 or 1 h, except for the unaffected *BrIPT5-1*. By contrast, the transcripts of *BrIPT9-1* was relatively steady, but *BrIPT2* and *BrIPT9-2* were also moderately induced by 6-BA. We hypothesized that this phenomenon could be attributed to the dynamic balance of various CKs in *B. rapa*. 6-BA is an iP type CK, and exogenous 6-BA application repressed the transcriptions of adenylate *IPT* genes, whereas the tRNA *IPT* genes were upregulated by a feedback regulation. A remarkable increase was observed in the levels of *BrCKX3-2* and *BrCKX5* transcripts after 6-BA treatment. Nevertheless, the expression levels of *BrCKX1-1*, -*1-2*, -*1-3*, -*2-2* and −*4* were decreased by various degrees. For the ABA treatment, five adenylate *IPT* genes in *B. rapa* were significantly repressed at 0.5 or 1 h, except for *BrIPT5-1*. Nevertheless, the expression of *BrIPT9-1* was relatively steady, whereas *BrIPT2* and *BrIPT9-2* was moderately induced by ABA, which was similar to the response patterns to 6-BA. The expressions of most of the *BrCKX* genes were down-regulated. However, the relative transcription levels of *BrCKX3-1*, *BrCKX*3-2, and *BrCKX5* were upregulated at 0.5 or 1 h. The antagonisms between CKs and ABA in plants may account for this phenomenon, but this mechanism has yet to be explored.

## Discussion

### The expansion and loss of *BrIPT* and *BrCKX* genes following hexaploidy formation by WGT

Brassicaceae genomes have undergone three rounds of whole genome duplication (WGD) and *Brassica* genomes have undergone another whole genome triplication (WGT) after speciation from *Arabidopsis thaliana*[36-38]. *B. rapa* is believed to have emerged from the *Brassica* progenitor approximately 8 MYA [33]. Considering WGT of the *Brassica* genomes, the *IPT* and *CKX* genes should have approximately three times as many members as that of *Arabidopsis*. However, we could only identify 13 *IPT* genes and 12 *CKX* genes in *B. rapa*, thereby suggesting a substantial loss of genes following hexaploidy formation by WGT. Gene loss was likewise observed for the *CRF* and *PG* gene families in *B. rapa* (unpublished data). Consistent with previous studies, the triplicated *B. rapa* genome contained only approximately twice the number of genes in *Arabidopsis* because of genome shrinkage [33]. Previous studies showed that *AtIPT6* is non-functional in the Wassilewskija (Ws) ecotype because of a frame-shift mutation [60], but is a functional gene in the Columbia (Col) ecotype [7]. By contrast, *AtIPT4* is a functional gene in all ecotypes [21]. Excluding a possible pseudogene, *AtIPT6*, the genomes of *A. lyrata*, and *A. thaliana* had equal numbers of *IPT* and *CKX* genes, thereby reflecting their relatively stable genome evolution because of a short separation time. However, both homologous genes of *AtIPT4* and *AtIPT6* in *B. rapa* were not found. Ando et al. [34] and O’Keefe et al. [35] identified several *BrIPT* and *BrCKX* genes, but still failed to obtain *BrIPT4* and *BrIPT6*. Thus, the homologous genes of *AtIPT4* and *AtIPT6* were probably lost in *B. rapa* during the long evolutionary history. Gene loss was a more common phenomenon in distantly related species. Le et al. [16] reported that *AtIPT1*, *AtIPT4*, *AtIPT6*, *AtIPT8,* and *GmIPT01* appeared to originate from a common ancestor. These genes expanded and were then retained in *Arabidopsis* but not in soybean after the speciation event. Genes with high homology to *AtIPT1*, *AtIPT4*, *AtIPT6*, and *AtIPT8* were also not found in rice [12], maize [14], and tomato [17]. We did not find homologs for *AtIPT4* and *AtIPT6* in this study. Nevertheless, genes homologous with *AtIPT1* and *AtIPT8* were identified in *B. rapa*, namely, *BrIPT1-1*, *BrIPT1-2*, *BrIPT8-1*, and *BrIPT8-2*. These genes all belonged to Group I with close relationships. Another piece of supporting evidence was that another *IPT* gene was found in *B. napus*, *BnIPT1*, which was homologous to *AtIPT1*[35]. The evolutionary mechanism of IPT genes in various species is still waiting to be clarified.

### The *IPT* and *CKX* genes in the bryophyte *P. patens*

The *IPT* and *CKX* genes have been discovered in the cyanobacteria *Nostoc* and the phytopathogen *Rhodococcus fascians*, respectively, with a large genetic distance from the other *IPT* and *CKX* genes. None of the *IPT* and *CKX* sequences have been found in green algae [15]. Schmuelling et al. [39] proposed that the presence of putative *CKX* sequences in cyanobacteria suggested that plant *CKX* genes might evolved from ancient chloroplast genes, which probably originated from the endosymbiosis of cyanobacteria. To date, the bryophyte *P. patens* is the earliest known terrestrial plant with *IPT* and *CKX* genes. Moreover, previous studies showed that the complete set of proteins in the CK signaling pathway first appeared in the said bryophyte [61]. Genes involved in the two-component system (TCS) of the *P. patens* were identified and characterized [62]. Thus, the CK metabolic pathway, and its signal transduction pathway imply that *P. patens* is the earliest species to have achieved a finely tuned control over CK homeostasis via biosynthesis that diverged to conquer land.

### Evolutionary patterns of *IPT* and *CKX* genes in the Cruciferae family

Since the tetraploidization of the *A. thaliana* ancestor 30–35 MYA, a wave of chromosomal rearrangements has modified its genome architecture. The majority of rearrangements had occurred before the *Arabidopsis*–*Brassica* split at 20–24 MYA; thus, the segmental architecture of the *A. thaliana* genome was predominantly conserved in *Brassica*[49,51,63]. We made a genome-level comparison with the three sequenced cruciferous species (*B. rapa*, *A. thaliana*, and *A. lyrata*). By analyzing the corresponding relationships of the *IPT* and *CKX* genes on chromosomes, traces of chromosomal rearrangement were easily captured. *Brassica* genomes have undergone whole genome triplication (WGT) after speciation from *Arabidopsis thaliana*, leading to significantly increased duplicated genes. Duplicated genes occur as the tandem duplicated genes and segmental duplicated genes. For the *IPT* and *CKX* genes in *B. rapa*, no tandem duplicated genes were found, but five pairs of segmental duplicates were found among 13 *BrIPT* genes, and three pairs of segmental duplicates were found among 12 *BrCKX* genes, suggesting that the expansion of *BrIPT* and *BrCKX* genes after speciation from *Arabidopsis thaliana* is mainly attributed to segmental duplication events during the whole genome triplication (WGT). The *K*_*a*_ (non-synonymous substitution rates) and *K*_*s*_ (synonymous substitution rates) is a measure to explore the mechanism of gene divergence after duplication. Large-scale duplication events are defined as simultaneous duplications of genes. Assuming a molecular clock, the synonymous substitution rates (*K*_*s*_) of these duplicates are expected to be similar over time. There are, however, substantial rate variations among genes [64]. We used the relative *K*_*s*_ measure as the proxy for time to evaluate the divergence time between *B. rapa* and *Arabidopsis.* The *K*_*s*_ values of the duplicated paralogs in *B. rapa* were notably much smaller than the values of the duplicated orthologous genes between *B. rapa* and *Arabidopsis*, further confirming that the duplicated *IPT* and *CKX* genes in *B. rapa* were produced during the whole genome triplication (WGT) after speciation from *Arabidopsis*.

### Changed expression patterns of the duplicated *BrIPT* and *BrCKX* genes

Duplication occurs in an individual and may be retained or lost in the population, similar to a point mutation [65]. Unless the presence of an extra amount of gene product is advantageous, two genes with identical functions are unlikely to be stably maintained in the genome. Theoretical population genetics predicates that both duplicates can be stably maintained when they differ in some aspects of their functions [66]. Sub-functionalization and neo-functionalization are two evolutionary fates of duplicated genes. These may account for the varied expression patterns of paralogs. Our study showed *BrIPT1-1* and *1–2*, as well as *BrCKX2-1, -2-2, -3-1, -3-2, -5, -7-1* and −*7-2*, were mainly expressed in the reproductive organs, whereas *BrIPT3-1, -3-2,* -*5-1* and −*5-2*, as well as *BrCKX4* and −*6* had major transcripts in the vegetative organs. Even the segmental duplicated gene pairs had inconsistent expression profiles. For instance, *BrCKX1-1* was mainly expressed in roots while *BrCKX1-2* maintained at high levels in stamens, flowers and petals. Meanwhile, *BrCKX1-3* was ubiquitously expressed, with relatively high levels in the petals and pistils. Likewise, a few duplicated gene pairs (*GmIPT08* and *GmIPT10*; *GmCKX05* and *GmCKX06*; *GmCKX12* and *GmCKX14*) have undergone expression divergence in soybean [16]. The response mechanisms of the two gene families varied for the different types of stresses. In this study, the *BrIPT5-1* was continuously induced by salt treatment, with 7-fold elevated expression levels. However, *BrCKX1-3* was suppressed, with dropped expression levels. The expressions of the majority of the *CKX* family in *Arabidopsis*, namely, *CKX2*, *CKX4*, *CKX5*, and *CKX7*, decreased with salt treatment; whereas expressions of the remaining three genes (*CKX1*, *CKX3* and *CKX6*) increased [30]. Meanwhile, increasing evidence indicated that the response to environmental stresses for plant *IPT* and *CKX* genes was tissue or organ-dependent. Stress induced *ZmCKX1* expressions in leaves, but it had an opposite effect in roots [31]. The induction levels in the V6-stage leaves of soybean were correlated with the age of the trifoliate leaves; the older the leaf, the higher the induction. *GmIPT09* and *GmIPT13* were significantly induced by drought in the V6-stage leaves; the degree of induction of these genes was higher in the younger trifoliate leaves [16]. All these proofs strongly supported the hypothesis that daughter genes originated from a common parental gene might adopt part of the functions of their parental gene [67-69] or acquire new functions [70].

### The possible roles of tRNA *IPT* genes in plants

Three tRNA *IPT* genes (*BrIPT2, BrIPT9-1*, and *BrIPT9-2*) were identified to have unique gene structures and expression patterns in *B. rapa*. *AtIPT2*, *AtIPT9*, *ZmIPT1*, *ZmIPT10*, *OsIPT9*, and *OsIPT10* likewise belong to this group of tRNA *IPT* genes. The tRNA *IPT* genes are responsible for the synthesis of cZ-type CKs. Based on the data from various bioassays [71,72], the tZ was found to be a bioactive substance whereas cZ was reported to have a weak biological effect. Moreover, the 35S-linked *AtIPT2* did not induce CK responses in calli when CKs were absent [21]. Despite the presumed inactivity of cZ as a free hormone, the presence of free cZ-type CKs in plant tissues has been repeatedly reported. Some plant species contain detectable levels of diverse cZ-type CKs, which are occasionally the predominant group of total CKs. Gajdosova et al. [9] hypothesized that the cZ-type CKs may function as delicate regulators of the CK responses in plants under growth-limiting conditions. Similar to *AtIPT2* and *AtIPT9*, *BrIPT2*, *BrIPT9-1*, and *BrIPT9-2*, were ubiquitously expressed. The tRNA *IPT* genes in *B. rapa* notably responded to adverse environmental stresses, thereby indicating that the cZ biosynthesized by tRNA *IPT* genes may be also involved in stress resistance.

## Conclusions

In summary, in this study we identified 13 *IPT* and 12 *CKX* genes in Chinese cabbage genome. The gene structures, conserved domains and phylogenetic relationships were analyzed. Duplications, evolutionary patterns and divergence of the *IPT* and *CKX* genes were investigated. We also characterized their expression patterns in various tissues and organs, their responses to abiotic stresses and exogenous phytohormones. To date, this is the first genome-wide study of the *IPT* and *CKX* gene families in *B. rapa*. Our results will help to enhance the understanding of the evolution of dynamic functions and fates for these *BrIPT* and *BrCKX* genes. Chinese cabbage is one of the most important vegetables and is widely cultivated. Unveiling the roles of *BrIPT* and *BrCKX* genes in plant growth, development and stress adaptation processes may help molecular breeders to develop economically important high-yielding and high-quality stress-tolerant crops in agriculture.

## Methods

### Identification of *IPT* and *CKX* gene families in *B. rapa* genome

The *Arabidopsis IPT* and *CKX* genes were used as seed sequences to search the *Brassica* Database (BRAD; version 1.1; http://brassicadb.org/brad/) and the NCBI database (http://www.ncbi.nlm.nih.gov). A total of 13 *BrIPT* and 12 *BrCKX* candidate genes were identified based on BLASTN and BLASTP searches with a cut-off expect value of 100. To ensure that no additional related genes were overlooked by the database search, the identified *BrIPT* and *BrCKX* genes were used as query sequences in additional multiple database searches to confirm the initial list of genes. Similarly, the *IPT* and *CKX* genes from *Arabidopsis lyrata*, *Populus trichocarpa*, and *Physcomitrella patens* were identified from the *A. lyrata* genome (version 1.0; http://genome.jgi-psf.org/Araly1/Araly1.info.html), the poplar genome assembly (version 1.1; http://genome.jgi-psf.org/Poptr1_1/Poptr1_1.home.html), and the *P. patens* genome assembly (version 1.1; http://genome.jgi-psf.org/Phypa1_1/Phypa1_1.info.html), respectively. The Pfam database (http://pfam.janelia.org/) [73] and the Conserved Domain Database (CDD) of the National Center for Biotechnology Information (NCBI) [74] were used to analyze all of the sequences that met the given requirements. Genes that did not contain the known conserved domains and motifs of the gene families were excluded from further analysis.

### Multiple sequence alignment and phylogenetic analysis

The online MEME software (http://meme.sdsc.edu/meme/meme.html) was used to identify the BrIPT and BrCKX motifs, with an expected *e*-value of less than 2×10^-30^[75,76]. Multiple sequence alignments were conducted using ClustalX (version 1.81) [77]. Phylogenetic analysis was performed using the neighbor-joining (NJ) method of the MEGA (version 5.0) program [78]. The confidence limits of each branch in the phylogenetic tree were assessed by 1000 bootstrap replications and expressed as percentage values.

### Physiological and biochemical property analysis of BrIPT and BrCKX proteins

The isoelectric point (pI) of the BrIPT and BrCKX proteins were predicted using Compute pI/Mwsoftware (http://www.expasy.ch/tools/pi_tool.html) [79]. Their subcellular localization was predicted by the TargetP software of the CBS database (http://www.cbs.dtu.dk/services/TargetP) [80]. The glycosylation sites were predicted using NetNGly (http://www.cbs.dtu.dk/services/NetNGlyc/) (unpublished database).

### *Cis*-element analysis of the putative promoter regions of *BrIPT* and *BrCKX* genes

To identify *cis*-elements in the promoter regions of *BrIPT* and *BrCKX* genes, the 2000 bp sequences upstream of the transcriptional start site of each *BrIPT* and *BrCKX* gene were chosen. These sequences were used to query the PLACE database (http://www.dna.affrc.go.jp/PLACE/) [81], and the putative *cis*-regulatory elements of the promoter sequences were identified.

### Chromosome mapping of the *IPT* and *CKX* genes in *B. rapa*, *A. thaliana,* and *A. lyrata*

Maps of the chromosomes were drawn using MS Office software, based on the location of each gene along the specific length of each chromosome. Tandem duplications were defined as genes located within 20 loci from each other [16]. Segmental duplications were identified by synteny analysis using an online tool of the Plant Genome Duplication Database (PGDD; http://chibba.agtec.uga.edu/duplication/) [82].

### Evolutionary analysis of homologous genes between *B. rapa* and *A. thaliana*

Synteny analysis of the *BrIPT* and *BrCKX* genes were performed online using PGDD (http://chibba.agtec.uga.edu/duplication/). The occurrence of duplication events and homologous genes divergence, as well as the selective pressure on duplicated genes, were estimated by calculating synonymous (*K*_s_) and non-synonymous substitutions (*K*_a_) per site between the homologous genes using the LOCUS SEARCH utility of PGDD. The divergence time was calculated using the neutral substitution rate of 1.5×10^-8^ substitutions per site per year for the chalcone synthase gene (*Chs*) [48].

### Plant growth and treatments

The plant materials (*B. rapa* ssp*. pekinensis* cv*.* Zhonghan No. 1) were grown at the experimental farm in Zhejiang University until flowering. Steps taken in growing the sampling plants were as follows: First, the seeds were sown on 10 Oct, 2012, and then were transplanted in the field outside after the seedlings with 5~6 pieces true leaves on 15 Nov, 2012. Subsequently, the seedlings were carried out with normal management for about 4 months. Finally, the plants produced flowers after bolting and materials were sampled on 10 Mar, 2013. The roots, floral stems, leaves, flowers, immature siliques, sepals, petals, stamens, pistils, little buds (<1.6 mm), medium-sized buds (1.6 mm to 2.8 mm), and big buds (>2.8 mm) were sampled to analyze the tissue- or organ-specific expression of the different genes. Total roots and main floral stems were chosen. For the leaves materials, young leaves at the second and third nodes from top of the main floral stems were chosen. For the flowers, new opened ones were selected. Immature siliques were defined as siliques about 3 days after pollination and fertilization. The diameters of the floral buds were measured using a Vernier caliper. All the materials were sampled from at least ten plants.

*B. rapa* ssp*. pekinensis* cv. Chiifu-401-42 plants were used in the different treatment conditions. All seedlings were grown under a 16 h/8 h light/dark photoperiod, at 25°C ± 1°C, for approximately 3 weeks. Only the second true leaves were sampled to minimize the differences between samples. The nutrient solution was supplemented with 200 mM NaCl for the salt treatments, and the leaves were collected at 0, 3, 8, and 16 h after stress induction. Water was withheld from three-week-old seedlings to initiate the drought treatment, and the leaves were subsequently classified into four levels, according to the degree of drought. Level 0 had normal leaves in well-watered seedlings. At level I (3 days after drought treatment), the leaves started to wither, whereas the leaves were severely withered at Level II (5 days after drought treatment). Level III (7 days after drought treatment) indicated that the whole seedlings had withered. The 3-week-old seedlings were sprayed with 100 μM 6-BA (6-Benzylaminopurine) for the cytokinin treatment and 100 μM ABA for the ABA treatment. The leaves were sampled at 0, 0.5, and 1 h after spraying, and the control was sprayed with double distilled water alone. All the materials sampled were immediately frozen in liquid nitrogen and stored in a refrigerator at −75°C.

### RNA extraction and qRT-PCR analysis

The total RNA was extracted using the TRIZOL reagent (Invitrogen, Germany), according to the manufacturer’s instructions. The first cDNA strand was generated using the Takara Reverse Transcription System (Japan) by following the manufacturer’s protocol. A maximum of 1 ug RNA was used for each reverse-transcription reaction. gDNA eraser in the kits was used to eliminate DNA to prevent DNA contamination. qRT-PCR was performed using the primers listed in Additional file 11. The primers were designed using the Primer (version 5.0) software. The specificity of each primer to their target genes was checked using the BLASTN program of BRAD. cDNA templates were firstly homogenized with the EASY Solution in the kits. Up to 2 μL of diluted cDNA were subjected to each qRT-PCR reaction for a final volume of 15 μL, which contained 7.5 μL SYBR Green Master Mix Reagent (Takara, Japan) and 0.3 μL specific primers (3 pmol). The qRT-PCR reactions were performed using a StepOne real-time PCR machine (Bio-RAD, USA), programmed to heat for 30 s at 95°C, followed by 40 cycles of 5 s at 95°C and 45 s at 53–58°C, and at the end, 1 cycle of 1 min at 95°C, 30 s at 50°C and 30 s at 95°C. The specificity of the reactions was verified by melting curve analysis and products were further confirmed by agarose gel electrophoresis. Two biological replicates were performed with three technical replicates for each sample plus the negative control. RNA for each biological replicate pooled from a number of individuals per treatment/tissue. The *BrActin1* gene was used as the reference gene. The comparative ΔΔ^CT^ method was used to calculate the relative expression levels of the different genes. The data of qRT-PCR were clustered using the average linkage method with Pearson correlation distance metric by Multiple Array Viewer [83].

## Competing interests

The authors declare that they have no competing interests.

## Authors’ contributions

ZNL carried out the experiment, analyzed the data, and drafted the manuscript. YXL, MZ, and YPL performed the qRT-PCR analysis. LJK and MHZ performed the abiotic stress analysis and wrote the manuscript. GL, JSC, and XLY proposed and supervised the research. All authors read and approved the final manuscript.

## Supplementary Material

Additional file 1 **Alignment of amino acid sequences of BrIPTs with AtIPTs. BrIPTs had two conserved regions, Region a and Region b.** Region a, denoted by red boxes, contained a putative ATP/GTP-binding sites (P-loop) motif (prosite PS00017: consensus sequence TGxGKS), which were underlined with a black line. Region b was a putative tRNA binding site, denoted by red boxes. Click here for file

Additional file 2 Summary of conserved motifs of BrIPT protein sequences. Click here for file

Additional file 4 **Alignment of amino acid sequences of BrCKXs with AtCKXs. The position of the motif for covalent binding of FAD cofactor (GHS, where H is the binding residue in all plant CKXs) was indicated.** Amino acid residues predicted based on the structure of ZmCKX1 to bind the cytokinin substrate were marked with an asterisk. The cytokinin N^9^ hydrogen-bonding residue that affected the substrate specificity was marked with a red arrow. Click here for file

Additional file 3 Summary of conserved motifs of BrCKX protein sequences. Click here for file

Additional file 5 **Synteny analysis of *BrIPT *genes in ±100 kb region with score greater than 1000.** Synteny analysis revealed evidence of the segmental duplications among *BrIPT* genes. Synteny analysis of *Bra036719* (*BrIPT2-1*) and *Bra040677* (*BrIPT2-2*) were omitted owing to unknown scaffold location of *Bra040677*. Click here for file

Additional file 6 **Synteny analysis of *BrCKX *genes in ±100 kb region with score greater than 1000.** Synteny analysis revealed evidence of the segmental duplications among *BrCKX* genes. Click here for file

Additional file 7 **Determination of *K***_***a ***_**and *K***_***s ***_**values of *IPT *genes.** The *K*_*a*_ and *K*_*s*_ values of *IPT* genes were determined between duplicated genes in *B. rapa* and the homologous genes among the *B. rapa*, *A. thaliana* and *A. lyrata*. — means no duplicated genes were found. Click here for file

Additional file 8 **Determination of *K***_***a ***_**and *K***_***s ***_**values of *CKX* genes.** The *K*_*a*_ and *K*_*s*_ values of *CKX* genes were determined between duplicated genes in *B. rapa* and the homologous genes among the *B. rapa*, *A. thaliana* and *A. lyrata*. - means no duplicated genes were found. Click here for file

Additional file 9 **Summary of the *cis*-elements found in the putative promoter regions of *BrIPT *genes. ***Cis*-elements with larger numbers were marked red. Click here for file

Additional file 10 **Summary of the *cis*-elements found in the putative promoter regions of *BrCKX *genes.***Cis*-elements with larger numbers were marked red. Click here for file

Additional file 11 Forward and Reverse Primers used in the qRT-PCR analysis. Click here for file
