# Supplementary material for: Identification, expression, and comparative genomic analysis of the IPT and CKX gene families in Chinese cabbage (Brassica rapa ssp. pekinensis)
Source: BMC Genomics. 2013 Aug 30;14:594. doi: 10.1186/1471-2164-14-594 (PMC3766048; doi:10.1186/1471-2164-14-594)
Supplement: Additional file 1 — Alignment of amino acid sequences of BrIPTs with AtIPTs. BrIPTs had two conserved regions, Region a and Region b. Region a, denoted by red boxes, contained a putative ATP/GTP-binding sites (P-loop) motif (prosite PS00017: consensus sequence TGxGKS), which were underlined with a black line. Region b was a putative tRNA binding site, denoted by red boxes. [file 1471-2164-14-594-S1.doc]

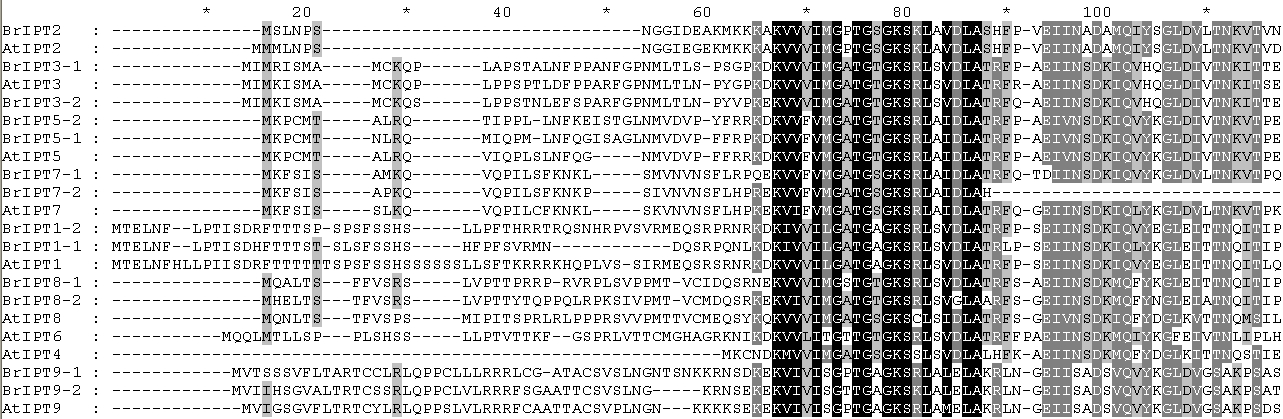


**Region a**


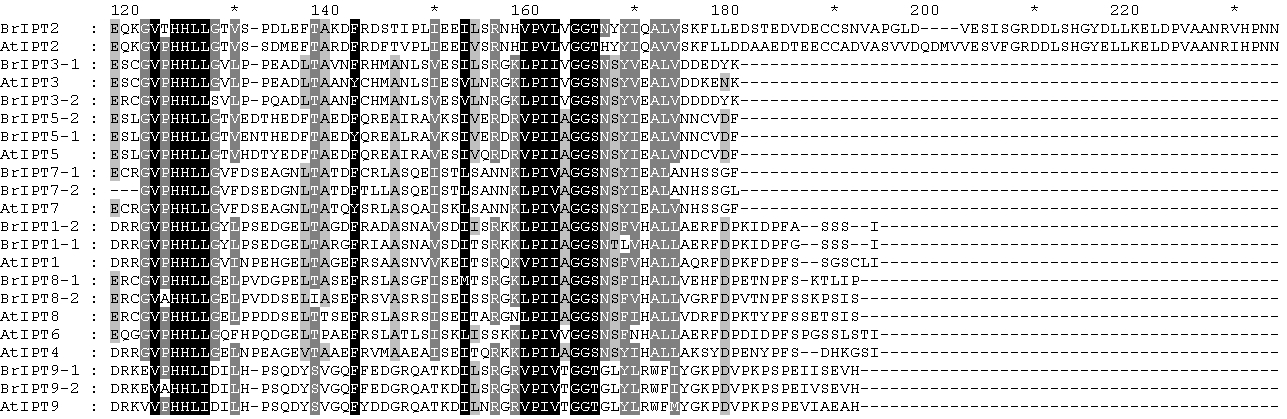


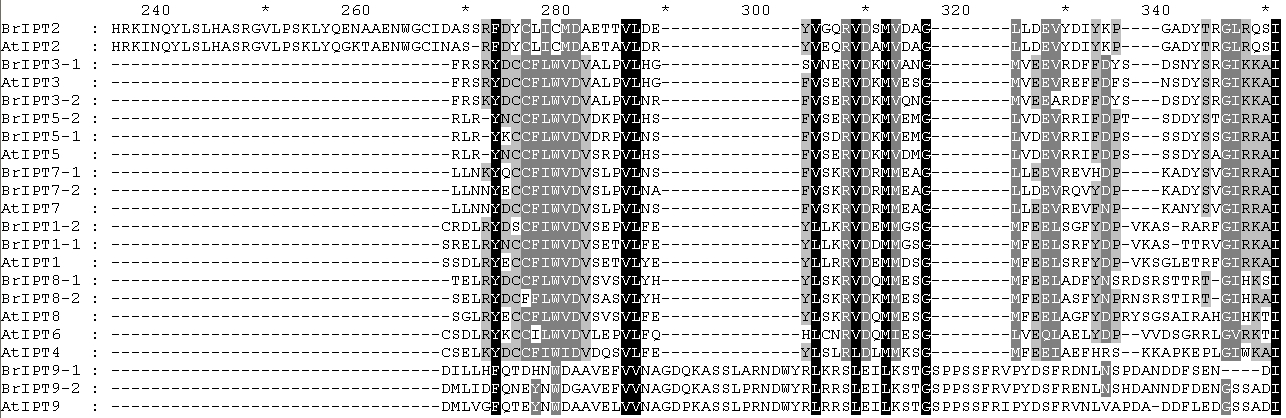


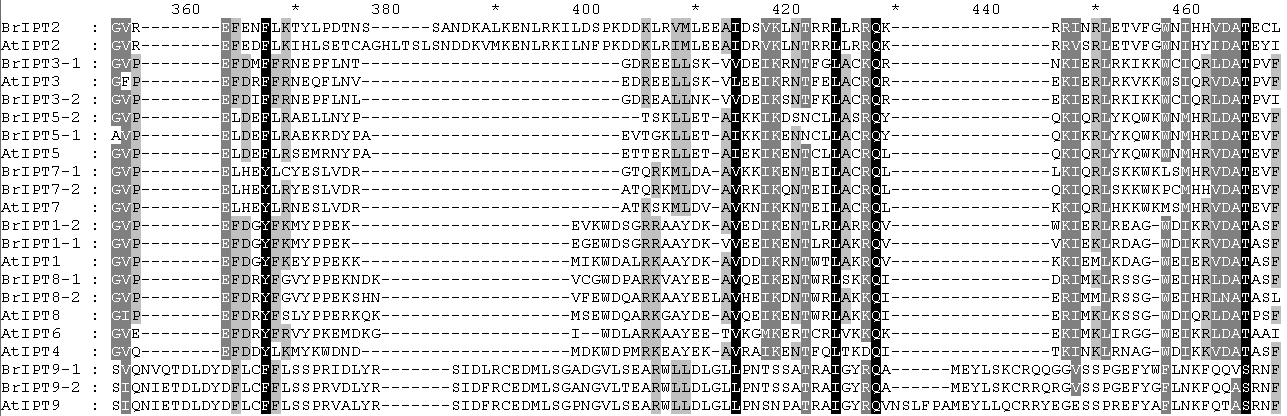


**Region b**


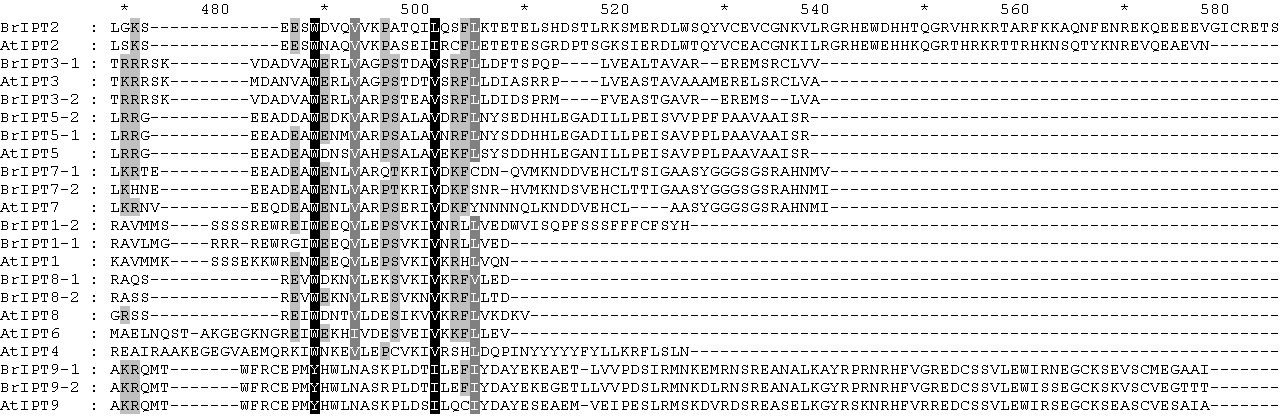


Additional file 1. Alignment of amino acid sequences of BrIPTs with AtIPTs. BrIPTs had two conserved regions, Region a and Region b. Region a, denoted by red boxes, contained a putative ATP/GTP-binding sites (P-loop) motif (prosite PS00017: consensus sequence TGxGKS), which were underlined with a black line. Region b was a putative tRNA binding site, denoted by red boxes.
