# Supplementary material for: Identification, expression, and comparative genomic analysis of the IPT and CKX gene families in Chinese cabbage (Brassica rapa ssp. pekinensis)
Source: BMC Genomics. 2013 Aug 30;14:594. doi: 10.1186/1471-2164-14-594 (PMC3766048; doi:10.1186/1471-2164-14-594)
Supplement: Additional file 4 — Alignment of amino acid sequences of BrCKXs with AtCKXs. The position of the motif for covalent binding of FAD cofactor (GHS, where H is the binding residue in all plant CKXs) was indicated. Amino acid residues predicted based on the structure of ZmCKX1 to bind the cytokinin substrate were marked with an asterisk. The cytokinin N9 hydrogen-bonding residue that affected the substrate specificity was marked with a red arrow. [file 1471-2164-14-594-S4.doc]

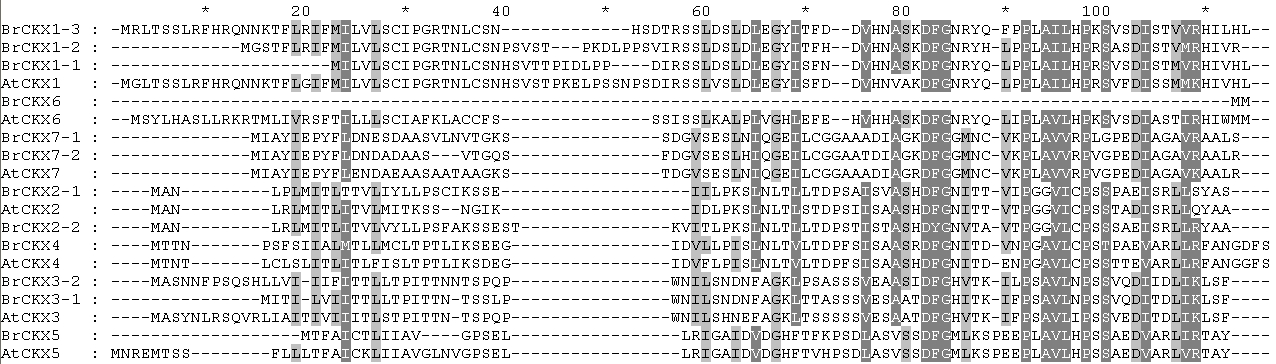


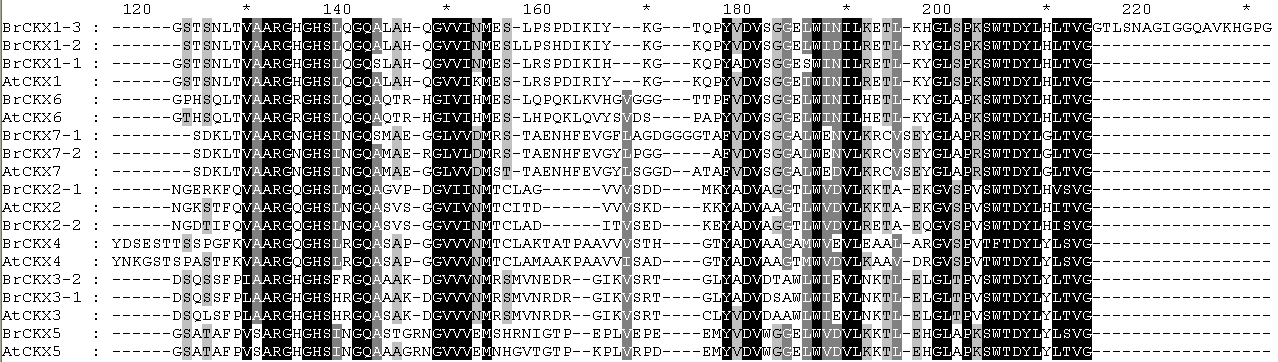


**FAD**


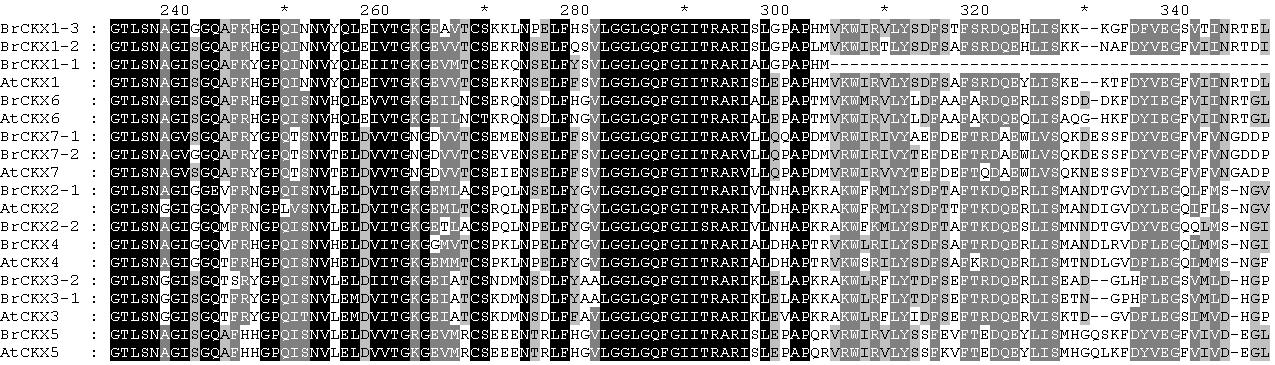


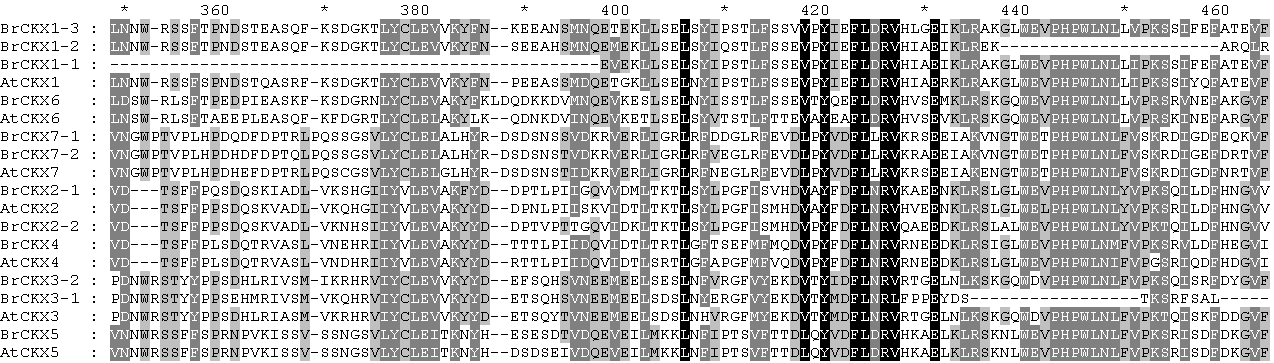


★

★

★


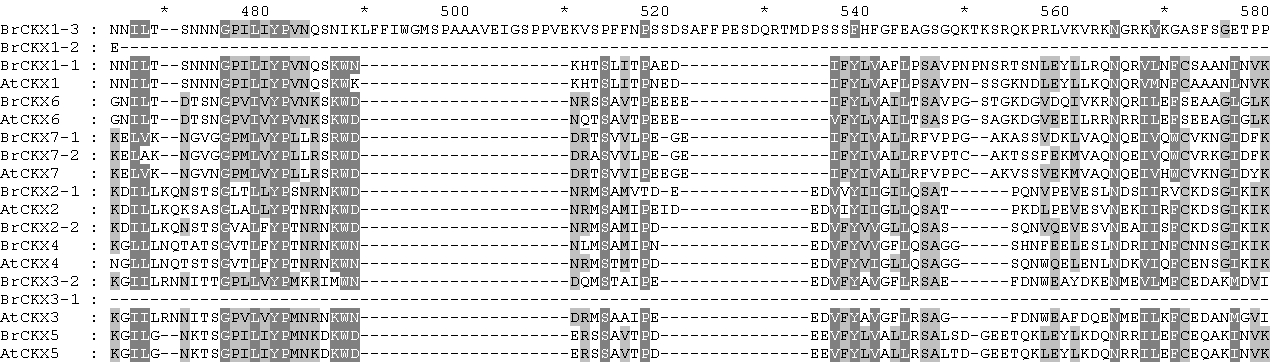


★

★

★

★


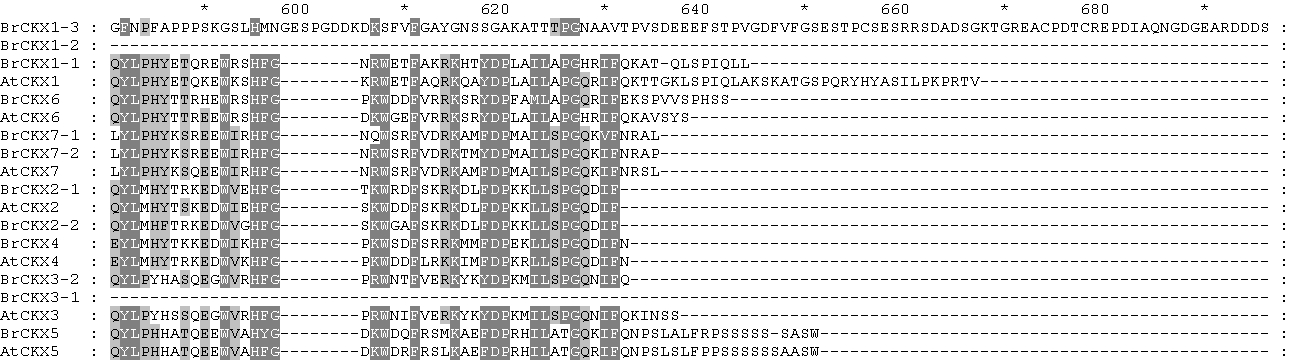


Additional file 3. Alignment of amino acid sequences of BrCKXs with AtCKXs. The position of the motif for covalent binding of FAD cofactor (GHS, where H is the binding residue in all plant CKXs) was indicated. Amino acid residues predicted based on the structure of ZmCKX1 to bind the cytokinin substrate were marked with an asterisk. The cytokinin N^9^ hydrogen-bonding residue that affected the substrate speciﬁcity was marked with a red arrow.
