# Supplementary material for: Identification, expression, and comparative genomic analysis of the IPT and CKX gene families in Chinese cabbage (Brassica rapa ssp. pekinensis)
Source: BMC Genomics. 2013 Aug 30;14:594. doi: 10.1186/1471-2164-14-594 (PMC3766048; doi:10.1186/1471-2164-14-594)
Supplement: Additional file 5 — Synteny analysis of BrIPT genes in ±100 kb region with score greater than 1000. Synteny analysis revealed evidence of the segmental duplications among BrIPT genes. Synteny analysis of Bra036719 (BrIPT2-1) and Bra040677 (BrIPT2-2) were omitted owing to unknown scaffold location of Bra040677. [file 1471-2164-14-594-S5.doc]

**
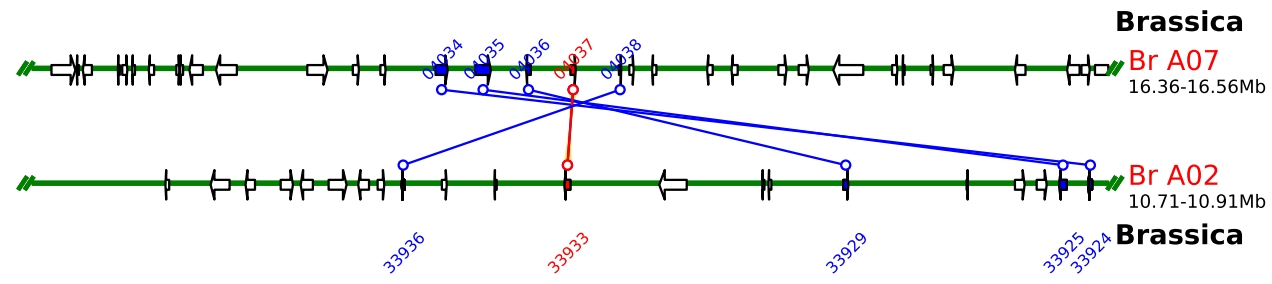
**

***BrIPT1-1***

***BrIPT1-2***

**
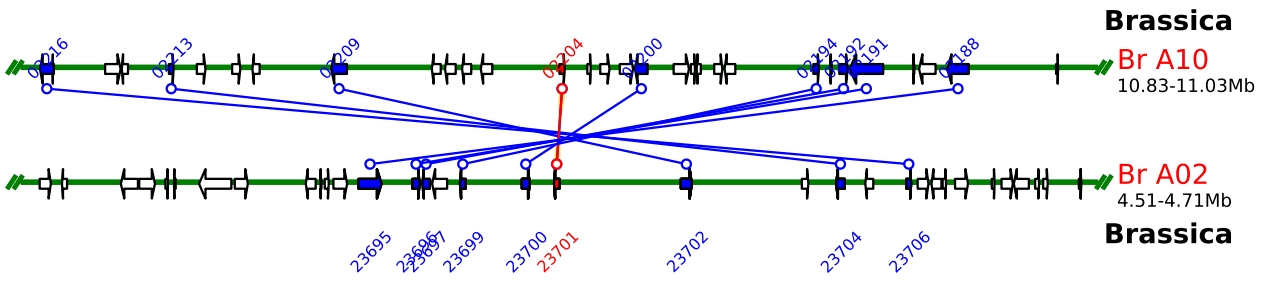
**

***BrIPT5-1***

***BrIPT5-2***

**
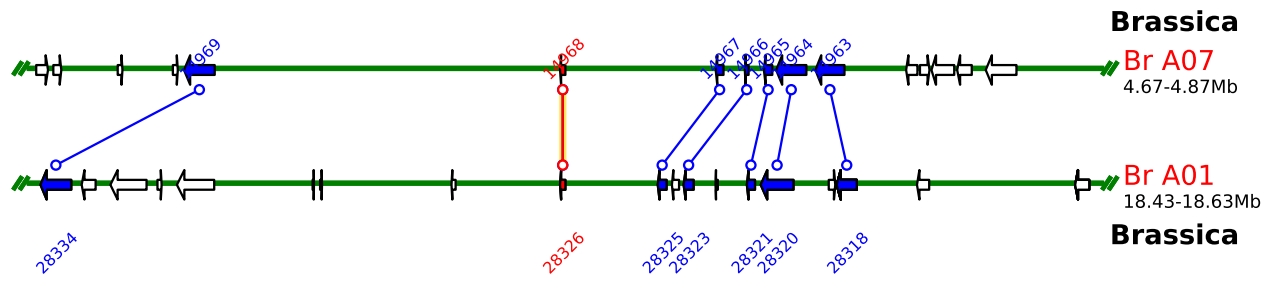
**

***BrIPT7-1***

***BrIPT7-2***

**
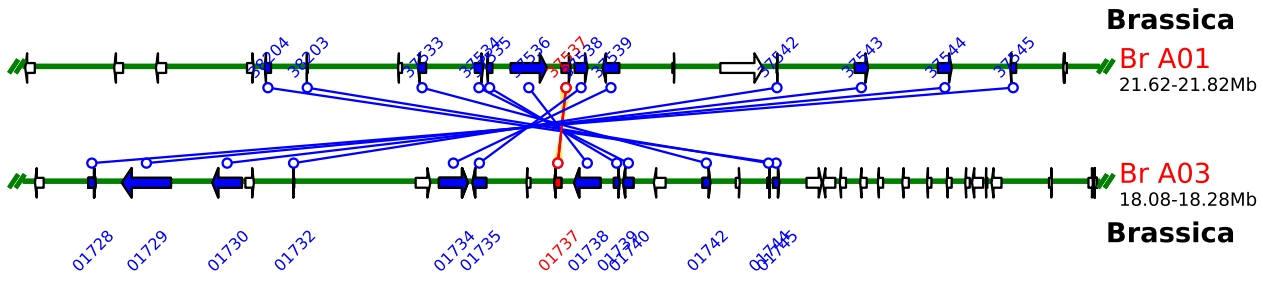
**

***BrIPT8-1***

***BrIPT8-2***

**
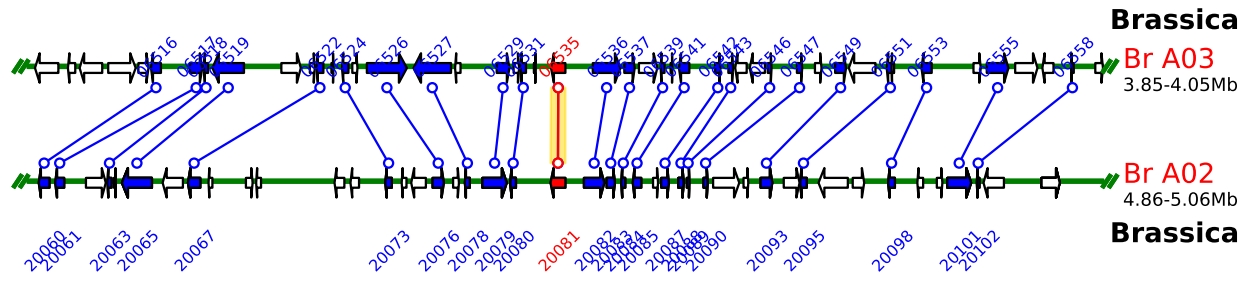
**

***BrIPT9-1***

***BrIPT9-2***

Additional file 5. Synteny analysis of *BrIPT* genes in ±100kb region with score greater than 1000. Synteny analysis revealed evidence of the segmental duplications among *BrIPT* genes. Synteny analysis of *Bra036719* (*BrIPT2-1*) and *Bra040677* (*BrIPT2-2*) were omitted owing to unknown scaffold location of *Bra040677*.
