# Supplementary material for: Identification, expression, and comparative genomic analysis of the IPT and CKX gene families in Chinese cabbage (Brassica rapa ssp. pekinensis)
Source: BMC Genomics. 2013 Aug 30;14:594. doi: 10.1186/1471-2164-14-594 (PMC3766048; doi:10.1186/1471-2164-14-594)
Supplement: Additional file 6 — Synteny analysis of BrCKX genes in ±100 kb region with score greater than 1000. Synteny analysis revealed evidence of the segmental duplications among BrCKX genes. [file 1471-2164-14-594-S6.doc]

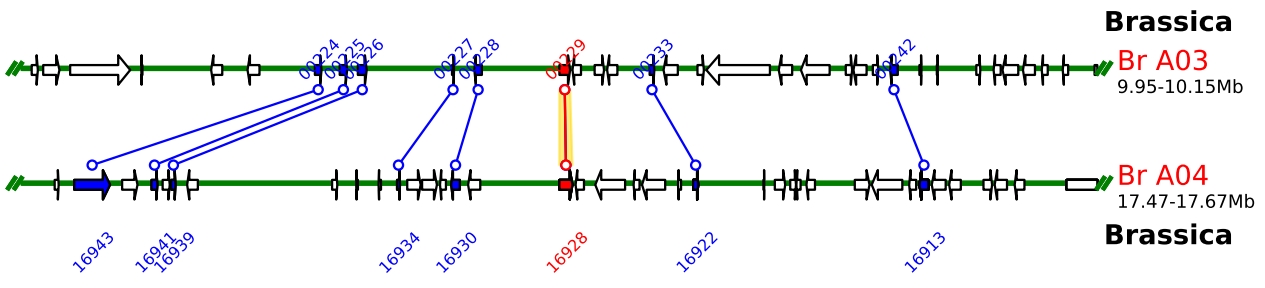


***BrCKX1-1***

***BrCKX1-2***


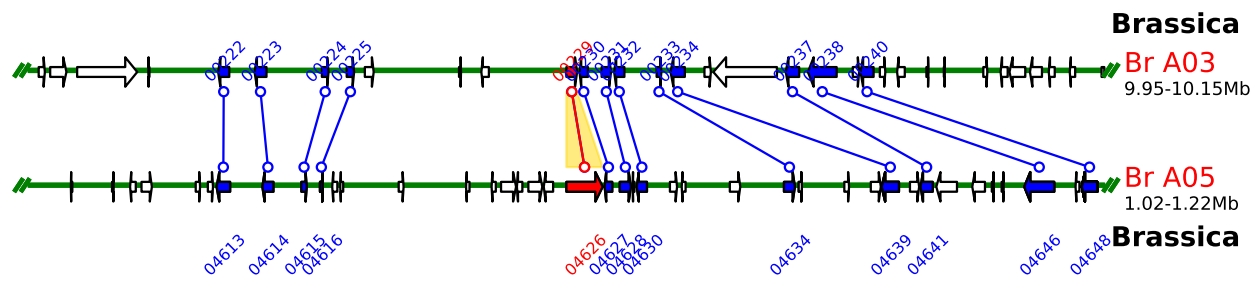


***BrCKX1-1***

***BrCKX1-3***


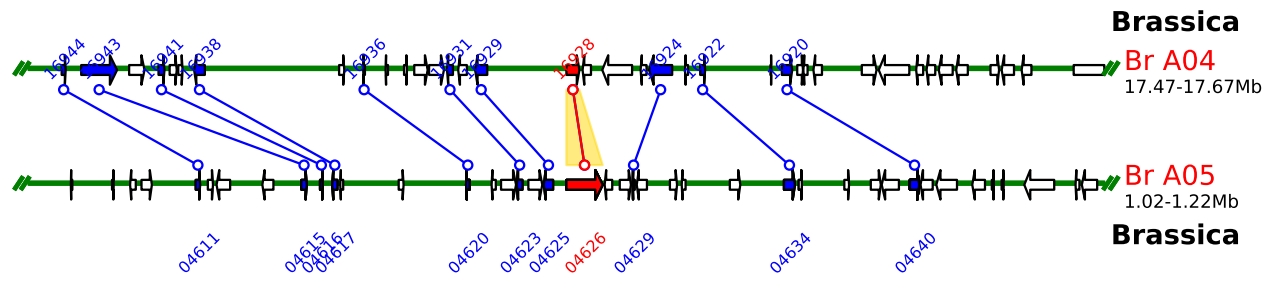


***BrCKX1-2***

***BrCKX1-3***


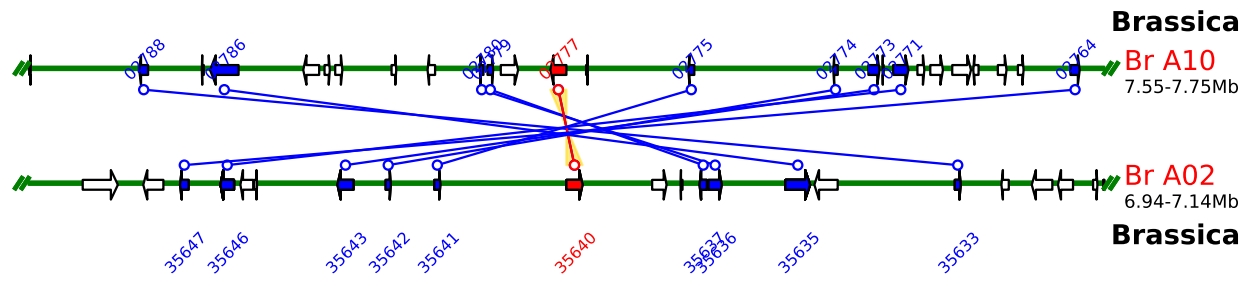


***BrCKX3-1***

***BrCKX3-2***


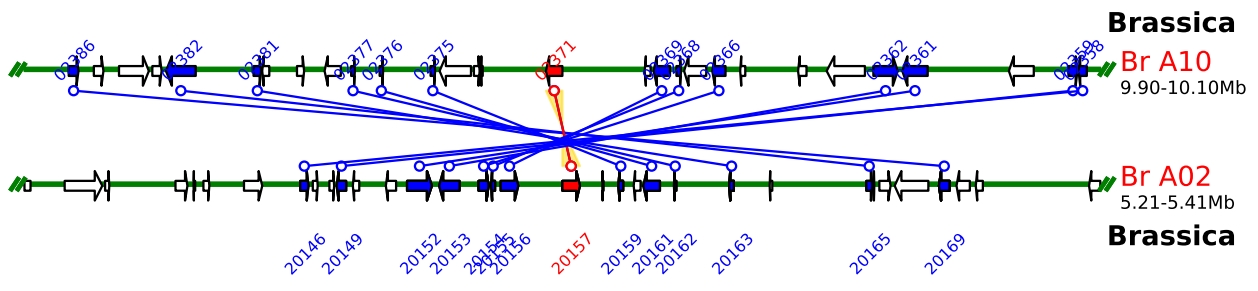


***BrCKX7-1***

***BrCKX7-2***

Additional file 6. Synteny analysis of *BrCKX* genes in ±100kb region with score greater than 1000. Synteny analysis revealed evidence of the segmental duplications among *BrCKX* genes.
