# Supplementary material for: Identification, expression, and comparative genomic analysis of the IPT and CKX gene families in Chinese cabbage (Brassica rapa ssp. pekinensis)
Source: BMC Genomics. 2013 Aug 30;14:594. doi: 10.1186/1471-2164-14-594 (PMC3766048; doi:10.1186/1471-2164-14-594)
Supplement: Additional file 7 — Determination of Ka and Ks values of IPT genes. The Ka and Ks values of IPT genes were determined between duplicated genes in B. rapa and the homologous genes among the B. rapa, A. thaliana and A. lyrata. — means no duplicated genes were found. [file 1471-2164-14-594-S7.doc]

| Gene name  Species | *BrIPT1-1*(Br004037)  Br A07 | | | *BrIPT1-2*(Bra033933)  Br A02 | | | *BrIPT2*(Bra034366)  Br A04 | | | *BrIPT3-1*(Bra040431)  Br A04 | | | *BrIPT3-2*(Bra007728)  Br A09 | | | *BrIPT5-1*(Bra002204)  Br A10 | | | *BrIPT5-2*(Bra023701)  Br A02 | | |
| --- | --- | --- | --- | --- | --- | --- | --- | --- | --- | --- | --- | --- | --- | --- | --- | --- | --- | --- | --- | --- | --- |
|  | Locus | *K_a_* | *K_s_* | Locus | *K_a_* | *K_s_* | Locus | *K_a_* | *K_s_* | Locus | *K_a_* | *K_s_* | Locus | *K_a_* | *K_s_* | Locus | *K_a_* | *K_s_* | Locus | *K_a_* | *K_s_* |
| *B. rapa* | Bra  033933  Br A02 | 0.08 | 0.36 | Br  004037  Br A07 | 0.08 | 0.36 | — | — | — | — | — | — | — | — | — | Bra  023701  Br A02 | 0.06 | 0.40 | Bra  002204  Br A10 | 0.06 | 0.40 |
| *A. thaliana* | AT1G  68460  At chr1 | 0.17 | 0.64 | AT1G  68460  At chr1 | 0.15 | 0.62 | AT2G  27760  At chr2 | 0.11 | 0.42 | AT3G  63110  At chr3 | 0.08 | 0.56 | AT3G  63110  At chr3 | 0.08 | 0.51 | AT5G  19040  At chr5 | 0.06 | 0.43 | AT5G  19040  At chr5 | 0.07 | 0.55 |
| *A. lyrata* | 16040709  Al sca2 | 0.16 | 0.62 | 16040709  Al sca2 | 0.14 | 0.62 | 16055118  Al sca4 | 0.11 | 0.43 | 16049097  Al sca5 | 0.08 | 0.53 | 16049097  Al sca5 | 0.08 | 0.45 | 16044366  Al sca6 | 0.07 | 0.50 | 16044366  Al sca6 | 0.07 | 0.60 |

| Gene name  Species | *BrIPT7-1*(Bra014968)  Br A07 | | | *BrIPT7-2*(Bra028326)  Br A01 | | | *BrIPT8-1*(Bra037537)  Br A01 | | | *BrIPT8-2*(Bra001737)  Br A03 | | | *BrIPT9-1*(Bra006535)  Br A03 | | | *BrIPT9-2*(Bra020081)  Br A02 | | |
| --- | --- | --- | --- | --- | --- | --- | --- | --- | --- | --- | --- | --- | --- | --- | --- | --- | --- | --- |
|  | Locus | *K_a_* | *K_s_* | Locus | *K_a_* | *K_s_* | Locus | *K_a_* | *K_s_* | Locus | *K_a_* | *K_s_* | Locus | *K_a_* | *K_s_* | Locus | *K_a_* | *K_s_* |
| *B. rapa* | Bra  028326  Br A01 | 0.06 | 0.33 | Bra  014968  Br A07 | 0.06 | 0.33 | Bra  001737  Br A03 | 0.12 | 0.34 | Bra  037537  Br A01 | 0.12 | 0.34 | Bra  020081  Br A02 | 0.06 | 0.35 | Bra  006535  Br A03 | 0.06 | 0.35 |
| *A. thaliana* | AT3G  23630  At chr3 | 0.08 | 0.54 | AT3G  23630  At chr3 | 0.09 | 0.46 | AT3G  19160  At chr3 | 0.16 | 0.47 | AT3G  19160  At chr3 | 0.16 | 0.57 | AT5G  20040  At chr5 | 0.11 | 0.41 | AT5G  20040  At chr5 | 0.10 | 0.36 |
| *A. lyrata* | 16045271  Al sca3 | 0.07 | 0.58 | 16045271  Al sca3 | 0.09 | 0.51 | 16046010  Al sca3 | 0.15 | 0.49 | 16046010  Al sca3 | 0.14 | 0.53 | 16052301  Al sca6 | 0.11 | 0.36 | 16052301  Al sca6 | 0.10 | 0.33 |

Additional file 7. Determination of *K_a_* and *K_s_* values of *IPT* genes. The *K_a_* and *K_s_* values of *IPT* genes were determined between duplicated genes in *B. rapa* and the homologous genes among the *B. rapa*, *A. thaliana* and *A. lyrata***.** — means no duplicated genes were found.
