# Supplementary material for: Identification, expression, and comparative genomic analysis of the IPT and CKX gene families in Chinese cabbage (Brassica rapa ssp. pekinensis)
Source: BMC Genomics. 2013 Aug 30;14:594. doi: 10.1186/1471-2164-14-594 (PMC3766048; doi:10.1186/1471-2164-14-594)
Supplement: Additional file 8 — Determination of Ka and Ks values of CKX genes. The Ka and Ks values of CKX genes were determined between duplicated genes in B. rapa and the homologous genes among the B. rapa, A. thaliana and A. lyrata. - means no duplicated genes were found. [file 1471-2164-14-594-S8.doc]

| Gene name  Species | *BrCKX1-1*(Bra000229)  Br A03 | | | *BrCKX1-2*(Bra16928)  Br A04 | | | *BrCKX1-3*(Bra004626)  Br A05 | | | *BrCKX2-1*(Bra036719)  Br A09 | | | *BrCKX2-2*(Bra040677)  Br sca232 | | | *BrCKX3-1*(Bra002777)  Br A10 | | | *BrCKX3-2*(Bra035640)  Br A02 | | |
| --- | --- | --- | --- | --- | --- | --- | --- | --- | --- | --- | --- | --- | --- | --- | --- | --- | --- | --- | --- | --- | --- |
|  | Locus | *K_a_* | *K_s_* | Locus | *K_a_* | *K_s_* | Locus | *K_a_* | *K_s_* | Locus | *K_a_* | *K_s_* | Locus | *K_a_* | *K_s_* | Locus | *K_a_* | *K_s_* | Locus | *K_a_* | *K_s_* |
| *B. rapa* | Bra  016928  Br A04 | 0.32 | 0.59 | Bra  000229  Br A03 | 0.32 | 0.59 | Bra  000229  Br A03 | 0.22 | 0.57 | — | — | — | — | — | — | Bra  035640  Br A02 | 0.08 | 0.30 | Bra  002777  Br A10 | 0.08 | 0.30 |
|  | Bra  004626  Br A05 | 0.22 | 0.57 | Bra  004626  Br A05 | 0.08 | 0.46 | Bra  016928  Br A04 | 0.08 | 0.46 | — | — | — | — | — | — | — | — | — | — | — | — |
| *A. thaliana* | — | — | — | AT2G  41510  At chr2 | 0.07 | 0.42 | AT2G  41520  At chr2 | 0.14 | 0.37 | AT2G  19500  At chr2 | 0.10 | 0.53 | AT2G  19500  At chr2 | 0.10 | 0.49 | AT5G  56970  At chr5 | 0.09 | 0.41 | AT5G  56970  At chr5 | 0.07 | 0.30 |
| *A. lyrata* | — | — | — | 16045246  Al sca4 | 0.07 | 0.42 | 16058210  Al sca4 | 0.13 | 0.35 | 16056506  Al sca3 | 0.10 | 0.48 | 16056506  Al sca3 | 0.10 | 0.47 | 16037470  Al sca8 | 0.08 | 0.39 | 16037470  Al sca8 | 0.07 | 0.31 |

| Gene name  Species | *BrCKX4*(Bra024135)  Br A03 | | | *BrCKX5*(Bra015842)  Br A07 | | | *BrCKX6*(007743)  Br A09 | | | *BrCKX7-1*(Bra002371)  Br A10 | | | *BrCKX7-2*(Bra020157)  Br A02 | | |
| --- | --- | --- | --- | --- | --- | --- | --- | --- | --- | --- | --- | --- | --- | --- | --- |
|  | Locus | *K_a_* | *K_s_* | Locus | *K_a_* | *K_s_* | Locus | *K_a_* | *K_s_* | Locus | *K_a_* | *K_s_* | Locus | *K_a_* | *K_s_* |
| *B. rapa* | — | — | — | — | — | — | — | — | — | Bra  020157  Br A02 | 0.04 | 0.34 | Bra  002371  Br A10 | 0.04 | 0.34 |
| *A. thaliana* | AT2G  19500  At chr2 | 0.21 | 0.9 | AT1G  75450  At chr1 | 0.03 | 0.47 | AT3G  63440  At chr3 | 0.08 | 0.53 | AT5G  21482  At chr5 | 0.05 | 0.51 | AT5G  21482  At chr5 | 0.05 | 0.50 |
|  | AT4G29740  At chr4 | 0.08 | 0.43 |  |  |  |  |  |  |  |  |  |  |  |  |
| *A. lyrata* | 16057552  Al sca7 | 0.09 | 0.41 | 16050634  Al sca2 | 0.04 | 0.43 | 16045116  Al sca5 | 0.08 | 0.51 | 16065897  Al sca6 | 0.04 | 0.47 | 16065897  Al sca6 | 0.05 | 0.49 |

Additional file 8. Determination of *K_a_* and *K_s_* values of *CKX* genes. The *K_a_* and *K_s_* values of *CKX* genes were determined between duplicated genes in *B. rapa* and the homologous genes among the *B. rapa*, *A. thaliana* and *A. lyrata***.** — means no duplicated genes were found.
