# Supplementary material for: Identification, expression, and comparative genomic analysis of the IPT and CKX gene families in Chinese cabbage (Brassica rapa ssp. pekinensis)
Source: BMC Genomics. 2013 Aug 30;14:594. doi: 10.1186/1471-2164-14-594 (PMC3766048; doi:10.1186/1471-2164-14-594)
Supplement: Additional file 9 — Summary of the cis-elements found in the putative promoter regions of BrIPT genes. Cis-elements with larger numbers were marked red. [file 1471-2164-14-594-S9.doc]

Additional file 9. Summary of the *cis*-elements found in the putative promoter regions of *BrIPT* genes. *Cis*-elements with larger numbers were marked red.

| Abiotic  Stress | Gene name  *cis*-element | ***BrIPT***  ***1-1*** | ***BrIPT***  ***1-2*** | ***BrIPT***  ***2*** | ***BrIPT***  ***3-1*** | ***BrIPT***  ***3-2*** | ***BrIPT***  ***5-1*** | ***BrIPT***  ***5-2*** | ***BrIPT***  ***7-1*** | ***BrIPT***  ***7-2*** | ***BrIPT***  ***8-1*** | ***BrIPT***  ***8-2*** | ***BrIPT***  ***9-1*** | ***BrIPT***  ***9-2*** |
| --- | --- | --- | --- | --- | --- | --- | --- | --- | --- | --- | --- | --- | --- | --- |
| Drought-stress | S000133 | 0 | 0 | 0 | 0 | 0 | 0 | 0 | 0 | 0 | 0 | 0 | 0 | 0 |
|  | S000153 | 0 | 0 | 0 | 0 | 0 | 1 | 0 | 2 | 0 | 4 | 1 | 1 | 1 |
|  | S000174 | 1 | 1 | 1 | 2 | 1 | 0 | 0 | 1 | 1 | 0 | 2 | 1 | 3 |
|  | S000175 | 0 | 0 | 0 | 0 | 0 | 0 | 0 | 0 | 0 | 1 | 2 | 0 | 0 |
|  | S000176 | 1 | 2 | 6 | 5 | 4 | 6 | 4 | 3 | 1 | 6 | 0 | 7 | 3 |
|  | S000177 | 0 | 1 | 0 | 2 | 1 | 0 | 1 | 2 | 0 | 1 | 0 | 0 | 0 |
|  | S000402 | 0 | 0 | 0 | 0 | 0 | 0 | 0 | 1 | 0 | 2 | 0 | 1 | 0 |
|  | S000408 | 4 | 5 | 4 | 8 | 2 | 4 | 3 | 3 | 4 | 2 | 6 | 7 | 4 |
|  | S000413 | 1 | 1 | 1 | 2 | 1 | 0 | 0 | 1 | 1 | 0 | 2 | 1 | 3 |
|  | S000414 | 0 | 3 | 0 | 0 | 2 | 3 | 0 | 3 | 0 | 2 | 2 | 1 | 0 |
|  | S000415 | 2 | 6 | 0 | 6 | 12 | 6 | 4 | 10 | 2 | 8 | 6 | 10 | 6 |
|  | S000418 | 0 | 0 | 0 | 0 | 0 | 0 | 0 | 2 | 0 | 2 | 0 | 1 | 0 |
| Salt-  stress | S000402 | 0 | 0 | 0 | 0 | 0 | 0 | 0 | 1 | 0 | 2 | 0 | 1 | 0 |
|  | S000418 | 0 | 0 | 0 | 0 | 0 | 0 | 0 | 3 | 0 | 2 | 0 | 1 | 0 |
|  | S000453 | 5 | 6 | 8 | 2 | 1 | 3 | 5 | 10 | 5 | 3 | 2 | 1 | 5 |
| Heat-  stress | S000030 | 2 | 6 | 3 | 6 | 4 | 5 | 10 | 2 | 2 | 3 | 2 | 2 | 8 |
|  | S000418 | 0 | 0 | 0 | 0 | 0 | 0 | 0 | 0 | 0 | 2 | 0 | 1 | 0 |
| Cold-  stress | S000153 | 0 | 0 | 0 | 0 | 0 | 1 | 0 | 2 | 0 | 4 | 1 | 1 | 1 |
|  | S000157 | 0 | 0 | 0 | 0 | 0 | 0 | 0 | 0 | 0 | 1 | 0 | 1 | 0 |
|  | S000402 | 0 | 0 | 0 | 0 | 0 | 0 | 0 | 1 | 0 | 2 | 0 | 1 | 0 |
|  | S000407 | 10 | 12 | 10 | 32 | 26 | 10 | 16 | 12 | 10 | 4 | 16 | 12 | 8 |
|  | S000418 | 0 | 0 | 0 | 0 | 0 | 0 | 0 | 2 | 0 | 2 | 0 | 1 | 0 |
| Wound-stress | S000159 | 0 | 0 | 2 | 0 | 0 | 0 | 0 | 0 | 0 | 0 | 0 | 0 | 0 |
|  | S000242 | 0 | 1 | 0 | 0 | 0 | 0 | 0 | 0 | 0 | 0 | 0 | 0 | 0 |
|  | S000244 | 0 | 0 | 0 | 0 | 0 | 0 | 0 | 1 | 0 | 1 | 0 | 0 | 0 |
|  | S000444 | 1 | 0 | 0 | 0 | 0 | 0 | 0 | 0 | 1 | 0 | 0 | 0 | 0 |
|  | S000457 | 3 | 1 | 2 | 5 | 4 | 1 | 2 | 4 | 3 | 3 | 5 | 4 | 8 |
