# Supplementary material for: Identification, expression, and comparative genomic analysis of the IPT and CKX gene families in Chinese cabbage (Brassica rapa ssp. pekinensis)
Source: BMC Genomics. 2013 Aug 30;14:594. doi: 10.1186/1471-2164-14-594 (PMC3766048; doi:10.1186/1471-2164-14-594)
Supplement: Additional file 10 — Summary of the cis-elements found in the putative promoter regions of BrCKX genes.Cis-elements with larger numbers were marked red. [file 1471-2164-14-594-S10.doc]

Additional file 10. Summary of the *cis*-elements found in the putative promoter regions of *BrCKX* genes. *Cis*-elements with larger numbers were marked red.

| Abiotic  stress | Gene name  *cis*-element | *BrCKX*  *1-1* | *BrCKX*  *1-2* | *BrCKX*  *1-3* | *BrCKX*  *2-1* | *BrCKX*  *2-2* | *BrCKX*  *3-1* | *BrCKX*  *3-2* | *BrCKX*  *4* | *BrCKX*  *5* | *BrCKX*  *6* | *BrCKX*  *7-1* | *BrCKX*  *7-2* |
| --- | --- | --- | --- | --- | --- | --- | --- | --- | --- | --- | --- | --- | --- |
| Drought-stress | S000133 | 0 | 0 | 0 | 0 | 0 | 0 | 0 | 0 | 2 | 0 | 0 | 0 |
|  | S000153 | 1 | 1 |  | 1 | 0 | 0 | 0 | 2 | 1 | 0 | 1 | 1 |
|  | S000174 | 1 | 1 | 1 | 0 | 1 | 2 | 1 | 0 | 0 | 1 | 0 | 2 |
|  | S000175 | 0 | 0 | 0 | 0 | 0 | 0 | 0 | 0 | 0 | 0 | 3 | 2 |
|  | S000176 | 2 | 6 | 2 | 6 | 1 | 3 | 5 | 2 | 5 | 6 | 10 | 4 |
|  | S000177 | 1 | 2 | 0 | 0 | 1 | 1 | 2 | 1 | 1 | 2 | 2 | 2 |
|  | S000402 | 0 | 0 | 0 | 0 | 0 | 0 | 0 | 1 | 1 | 0 | 0 | 0 |
|  | S000408 | 4 | 3 | 3 | 5 | 7 | 7 | 5 | 1 | 3 | 3 | 10 | 6 |
|  | S000413 | 1 | 1 | 1 | 0 | 1 | 2 | 1 | 0 | 0 | 1 | 0 | 2 |
|  | S000414 | 0 | 0 | 2 | 1 | 2 | 2 | 2 | 3 | 14 | 3 | 0 | 0 |
|  | S000415 | 4 | 2 | 6 | 6 | 8 | 6 | 2 | 10 | 28 | 4 | 6 | 0 |
|  | S000418 | 0 | 1 | 0 | 0 | 0 | 0 | 0 | 2 | 1 | 0 | 0 | 0 |
| Salt-  stress | S000402 | 0 | 0 | 0 | 0 | 0 | 0 | 0 | 1 | 1 | 0 | 0 | 0 |
|  | S000418 | 0 | 1 | 0 | 0 | 0 | 0 | 0 | 2 | 1 | 0 | 0 | 0 |
|  | S000453 | 5 | 5 | 3 | 2 | 3 | 4 | 5 | 5 | 6 | 4 | 8 | 8 |
| Heat-  stress | S000030 | 9 | 9 | 9 | 4 | 5 | 8 | 6 | 5 | 4 | 5 | 3 | 8 |
|  | S000418 | 0 | 1 | 0 | 0 | 0 | 0 | 0 | 1 | 1 | 0 | 0 | 0 |
| Cold-  stress | S000153 | 1 | 1 |  | 1 | 0 | 0 | 0 | 2 | 1 | 0 | 1 | 1 |
|  | S000157 | 0 | 0 | 0 | 0 | 0 | 0 | 0 | 0 | 0 | 0 | 0 | 0 |
|  | S000402 | 0 | 0 | 0 | 0 | 0 | 0 | 0 | 1 | 1 | 0 | 0 | 0 |
|  | S000407 | 6 | 8 | 14 | 16 | 6 | 16 | 24 | 12 | 14 | 20 | 14 | 8 |
|  | S000418 | 0 | 1 | 0 | 0 | 0 | 0 | 0 | 2 | 1 | 0 | 0 | 0 |
| Wound-stress | S000159 | 0 | 0 | 0 | 0 | 0 | 0 | 0 | 0 | 0 | 0 | 0 | 0 |
|  | S000242 | 0 | 0 | 0 | 0 | 0 | 0 | 0 | 0 | 0 | 0 | 0 | 0 |
|  | S000244 | 0 | 0 | 0 | 0 | 0 | 0 | 0 | 0 | 1 | 0 | 0 | 0 |
|  | S000444 | 0 | 0 | 0 | 0 | 0 | 0 | 1 | 0 | 0 | 0 | 0 | 0 |
|  | S000457 | 7 | 6 | 7 | 9 | 6 | 4 | 5 | 4 | 11 | 5 | 3 | 0 |
