# Supplementary material for: Identification, expression, and comparative genomic analysis of the IPT and CKX gene families in Chinese cabbage (Brassica rapa ssp. pekinensis)
Source: BMC Genomics. 2013 Aug 30;14:594. doi: 10.1186/1471-2164-14-594 (PMC3766048; doi:10.1186/1471-2164-14-594)
Supplement: Additional file 11 — Forward and Reverse Primers used in the qRT-PCR analysis. [file 1471-2164-14-594-S11.doc]

Additional file 11. Forward and Reverse Primers used in the qRT-PCR analysis.

| Primer name | Forward primer sequence | Reverse primer sequence |
| --- | --- | --- |
| *BrActin1* | CGCTTAACCCGAAAGCTAAC | TACGCCCACTAGCGTAAAG |
| *BrIPT1-1* | GTTACTTCAAAATGTACCCACCG | TCTTCTTCTTCTTCCCATCAAAA |
| *BrIPT1-2* | ACGATAAGGCGGTGGAGG | TGGAAAATGGCTGGGAGATA |
| *BrIPT2* | AGAAGGCGAAGGTGGTGG | ATTGCGTCGGCGTTTATT |
| *BrIPT3-1* | TCACGAGGCGAAGGTCAA | CGACATCTCACGCTCCCT |
| *BrIPT3-2* | AAGGCGTAGATCAAAGGTGG | AACATTCGAGGGCTGTCAAT |
| *BrIPT5-1* | GACGAGGTTCGCCGCATCT | CACCTCCGCCGGATAATCACGCTTC |
| *BrIPT5-2* | GCGGGAAGCAATCAGAGC | TTCGACGTCGGGTAATTTAGCAGTT |
| *BrIPT7-1* | CCGACAAGATCCAAGTTTACA | CCGAGCAAGTGGTGAGGC |
| *BrIPT7-2* | ATCGACCTTGCACACGGCGTGCCTC | ACGCAAGGAGAGTGAAGTCGGTGGC |
| *BrIPT8-1* | ATCTTTGGCGTCAGGGTTCATCTCT | AACTCGGTAGGTATCAAGGTTTTAG |
| *BrIPT8-2* | TACACACAACCACCTCAACTCCGAC | GCTCCCATTATGACAATCACTTT |
| *BrIPT9-1* | CGAATAAGAAGAGAAACTCCGATAA | CGATAAGATGATGAGGCA |
| *BrIPT9-2* | ATTCTTAGTAGAGGGCGTGT | CAGCAGATGAACCATTCTCATCAAA |
| *BrCKX1-1* | GGTTCCACATCCCTGGCTGAATCTC | GGACCGTTGTTGTTGCTTG |
| *BrCKX1-2* | TCCTTCTGTTAGTACCCCAAAAGAT | GAGGTGGTAAGTGGTATCTGTTGCC |
| *BrCKX1-3* | TCTTCAACCCTTCTTCCGACTC | AGGCACCTTTCACCTTTCTACC |
| *BrCKX2-1* | GCGTAGTGGTTTCCGATGA | GCATTTGACAGTGTTCCTCC |
| *BrCKX2-2* | AGCACTAAAGTTATTACTCTAC | GTTGATAACCACTCCACCTGA |
| *BrCKX3-1* | GAAACTTCCCAACACTCAGTCAAC | TTACAACGCAGAGAAACGCGA |
| *BrCKX3-2* | GGTAGGGTTCTTACGATCTGC | TTCCACCTCGGACCAAAA |
| *BrCKX4* | TCGCCAAGACGGCTACAC | CCTCAAGAACCTCCACCCAC |
| *BrCKX5* | TTTCTACCTCGTGGCTTTACTG | CCACTCCTCTTGCGTTGC |
| *BrCKX6* | GAAGCAGCAGGTTTAGGGC | CAAGCATTGCGAAGGGAT |
| *BrCKX7-1* | GGCCGGTGACGGTGGTGGTGGTACG | TAACGGAACGCTTGTCCACTAACGC |
| *BrCKX7-2* | ATTTACCCGGCGGGGCGT | CGTCAACCCGAGGTAATCAGT |
